# Computational insights into the physico-chemical properties of pure and single-atom copper–indium sub-nanometre clusters: a DFT-genetic algorithm approach†

**DOI:** 10.1039/d4ra07404a

**Published:** 2025-02-20

**Authors:** Norah O. Alotaibi, Heider A. Abdulhussein, Shatha M. Alamri, Noorhan Ali Hamza, Abbas H. Abo Nasria

**Affiliations:** a Chemistry Department, Faculty of Science, King Abdulaziz University Jeddah 21589 Saudi Arabia; b Department of Chemistry, Faculty of Science, University of Kufa Najaf Iraq haydera.abdulhussein@uokufa.edu.iq; c College of Engineering, University of Warith Al-Anbiyaa Kerbala Iraq heider.abd@uowa.edu.iq; d Department of Chemistry, Faculty of Education for Pure Science, University of Kerbala Kerbala Iraq; e Department of Physics, Faculty of Science, University of Kufa Najaf Iraq

## Abstract

Catalysis involving Cu–In nanoparticles represents an exciting area of technological advancement. However, our fundamental grasp of the mechanisms governing mixing within Cu–In clusters at the sub-nanometer scale and their gas-phase physicochemical properties remains inadequate. We have determined the global minima for gas-phase Cu–In clusters containing 3 to 10 atoms using the Mexican Enhanced Genetic Algorithm in conjunction with density functional theory. Simulations were also conducted for Cu and In atoms and their dimers at the same theoretical level. Comparative analyses were performed between mixed Cu–In systems and their pure counterparts, with pure Cu and In clusters being modeled up to 13 atoms. The findings indicate a 2D–3D transition for pure Cu clusters at 7 atoms, while for pure In clusters, this transition occurs at 5 atoms. For Cu–In nanoalloys, both cluster size and doping have been found to significantly and non-linearly impact cluster structures. Stability assessments, including binding energies, second differences in energy, and mixing energies, were used to evaluate the energetics, structures, and segregation tendencies of sub-nanometer Cu–In clusters. The most stable composition, as indicated by mixing energies, is achieved when the Cu to In ratio is equal or nearly equal. The HSE06 spin-projected band structure reveals that In_4_Cu_1_ displays magnetic properties akin to monometallic In_5_. Conversely, the spin-projected band structure and partial density of states (PDOS) analysis for bimetallic Cu_7_In_1_ show that the cluster is non-magnetic. Analysis of the topological parameters of Cu–Cu, In–In, and Cu–In bonds in bimetallic clusters, using the Quantum Theory of Atoms in Molecules (QTAIMs), indicates that these interactions are not purely closed-shell but involve significant covalent contributions.

## Abstract 2

This study explores the structural, segregation, electronic, and energetic properties of pure and mono-substituted Cu–In sub-nanometer clusters using Mexican Enhanced Genetic Algorithm based DFT approach.

## Graphical abstract

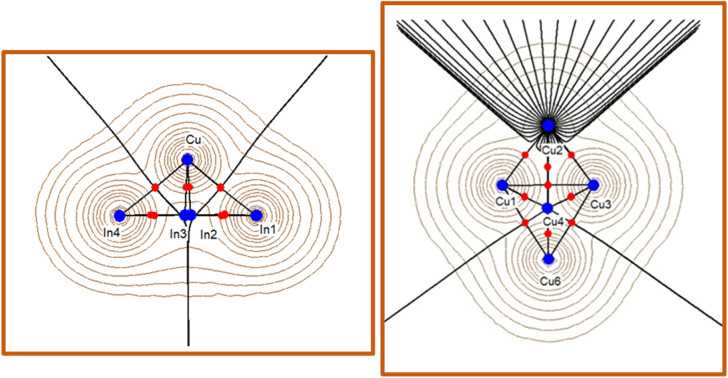

## 1. Introduction

Nanoparticles (NPs) are crucial in bridging the gap between bulk materials and small molecules^1^ due to their size-dependent properties, high surface-to-volume ratios, and quantum-size effects.^2^ These properties make metallic nanoparticles (MNPs) particularly attractive for various applications, including nanocatalysis, nanobiology, and nanodevices. By controlling the size, morphology, and composition of these nanoparticles, researchers have achieved significant improvements in their optical,^3^ electronic,^4^ magnetic, and catalytic properties.^5^ This versatility has positioned NPs as pivotal players in addressing pressing energy and environmental challenges.^6–8^

Catalysis, especially at the nanoscale, is central to the development of high-performance materials with enhanced activity and selectivity.^9–13^ Metallic nanoparticles, particularly bimetallic alloys, have emerged as superior catalysts compared to their monometallic counterparts due to their tunable electronic and structural properties.^14–17^ Among the bimetallic systems, Cu-based alloys have garnered significant attention due to copper's earth-abundance, low cost,^18^ and versatility in catalytic applications, including CO_2_ reduction reactions (CO_2_RR).^19–25^ However, the catalytic performance of monometallic Cu is often limited by its high overpotential and competitive hydrogen evolution reaction (HER).^26^ Introducing a secondary metal, such as indium (In), offers an avenue to overcome these limitations by modulating the electronic structure and surface properties of Cu, thereby enhancing selectivity and activity.^24^

Doping is a powerful strategy for modifying the catalytic properties of metal clusters. The inclusion of dopant atoms fundamentally transforms the electronic structure, surface characteristics, and chemical reactivity of the host metal.^27^ This transformation often leads to enhanced catalytic performance, as doping can stabilize specific geometries, introduce new active sites, and modulate electronic density to optimize reaction pathways.^28–31^ Studies have demonstrated that doping can result in the redistribution of electron density, altering the charge states of active sites and facilitating stronger or more selective adsorption of reactants.^32^ The interplay between electronic and structural properties in doped clusters was meticulously analyzed by Barrabés *et al.*,^33^ who highlighted how alloying metals enhances both stability and activity by reducing surface strain and optimizing electronic interactions. Research by Manzoor *et al.*,^34^ and Dar *et al.*,^35^ has further illuminated the dual role of surface and electronic modifications in tailoring catalytic behavior. Beyond structural and electronic factors, thermodynamic studies have shown that doping can significantly influence the stability and reactivity of metal clusters. Researches^28,36^ emphasized the importance of compositional tuning to achieve optimal stability and catalytic efficiency, showing that doped systems often exhibit unique synergistic effects that are absent in their pure counterparts. These findings collectively underline the critical role of doping in advancing the design of next-generation catalysts with unparalleled performance and stability.

Indium is particularly well-suited as a dopant for Cu-based clusters due to several key attributes. Its non-toxic nature and relative abundance make it a sustainable choice for catalytic applications, particularly in energy and environmental sectors.^37^ Indium exhibits a unique combination of physical and chemical properties, including high electrical conductivity, low melting point, and exceptional chemical stability, which are crucial for enhancing catalytic efficiency.^38^ One of the most notable characteristics of indium is its strong binding affinity for key intermediates in CO_2_RR, particularly *HCOO, which promotes the selective formation of formate.^39^ This selectivity is critical in minimizing competing reactions, such as HER, which often reduce the overall efficiency of CO_2_RR.^37,40–42^ Indium's larger atomic radius compared to copper reduces surface energy, encouraging the occupation of low-coordination sites. These low-coordination sites are often more catalytically active, as they provide unique adsorption geometries and electronic environments conducive to efficient catalytic processes.^30,31,43^

The doping of Cu clusters with In has a profound effect on their reactivity by modifying their electronic and surface characteristics. Studies have shown that indium incorporation significantly lowers the reaction barrier for CO_2_ reduction by enhancing the stabilization of reaction intermediates, such as *COOH and *HCOO, on Cu surfaces.^44^ The electron density redistribution caused by In doping increases the nucleophilic character of Cu clusters, facilitating the adsorption and activation of CO_2_ molecules. This is consistent with findings that bimetallic systems exhibit improved charge transfer dynamics, enabling efficient catalytic pathways.^45^ By tailoring the interaction between active sites and reactants, In-doped Cu clusters achieve enhanced selectivity and activity, particularly for CO_2_-to-formate conversion. The doping also enhances the adsorption of *CO intermediates, crucial for C–C coupling reactions, by modifying the surface electronic structure through synergistic interactions between In and Cu. This results in higher yields of C_2_^+^ products, as demonstrated by the Cu_100_In_5.1_-CP catalyst reported recently by Han and his team,^46^ which achieved a maximum faradaic efficiency of 85.11% and a partial current density of 36.3 mA cm^−2^. Additionally, the atomic dispersion of In prevents aggregation, maintaining a high surface area and improving catalyst stability. Raman spectroscopy and XPS analyses achieved by the researchers revealed that In doping optimizes the surface properties of Cu clusters, increasing their electrochemical activity and facilitating stronger binding of intermediates, making them highly effective for CO_2_ reduction. These effects illustrate the crucial role of In doping in advancing the reactivity and efficiency of Cu-based catalysts.

In addition to theoretical insights, the synthesis of Cu–In clusters is a critical aspect to consider for practical applications. One of the most effective synthesis methods for bimetallic clusters involves the use of colloidal chemistry techniques.^47^ This approach allows for precise control over particle size, composition, and morphology, which are crucial for optimizing catalytic performance. For instance, the study by He *et al.*^48^ demonstrated the synthesis of bimetallic nanoparticles *via* a seed-mediated growth strategy. This method provides a robust framework for producing Cu–In clusters with tailored properties, enabling systematic investigations into their catalytic behavior. Wet chemical reduction and galvanic replacement are alternative techniques that can be employed to synthesize bimetallic clusters with homogeneous compositions.^49^ Liu *et al.*,^50^ explored galvanic replacement to create core–shell structures with excellent catalytic activity. Another promising approach involves microwave-assisted synthesis, as demonstrated by Som *et al.*,^51^ which offers rapid and energy-efficient production of bimetallic clusters. These methods provide the versatility required to explore a wide range of compositions and structures, aligning well with the goals of advancing CO_2_RR technologies.

On the energy landscape, a substantial number of global optimization algorithms have been demonstrated to identify locally stable structures,^52–54^ in other words, the global minimum (GM) and the low-lying minimum (LM). In evolutionary algorithms, genetic algorithms (GAs) are examples of algorithms that simulate and mimic the natural process of evolution.^55^ An array of systems ranging from zero- to three-dimensional systems have been predicted using GAs.^56^ To the best of our knowledge, no global optimization calculations have been published for single-atom copper–indium and pure indium nanoclusters, despite the number of researches achieved on their structure and properties.^38,57^ In this study, we utilize a Mexican Enhanced Genetic Algorithm (MEGA) coupled with density functional theory (DFT)^28,31,58,59^ to explore the global optimization of single-atom Cu–In clusters. By focusing on clusters ranging from pure Cu and In to monosubstituted compositions, we aim to uncover the structural characteristics, stability, and electronic properties of these nanoalloys. MEGA was chosen for global optimization due to its robustness in exploring large configurational spaces and efficiently locating global minima. The algorithm's crossover and mutation operations, coupled with energy-based fitness criteria, ensured a comprehensive search for stable cluster configurations.^28,31,58,59^ Our results provide valuable insights into the mechanisms of mixing and doping, revealing the impact of composition on the physicochemical properties of Cu–In clusters. This work not only bridges a significant gap in the literature but also highlights the potential of Cu–In clusters as efficient and selective catalysts for sustainable applications.

## 2. Methodology

### 2.1. Computational details

In this study, global optimizations are carried out using the Mexican Enhanced Genetic Algorithm (MEGA)^52,58,59^ utilizing an interface to the Vienna *Ab initio* Simulation Package (VASP)^60–63^ to simulate copper–indium nanoclusters and their monometallic counterparts. A parallel approach to DFT optimization is employed by utilizing multiple processors. By employing a pool methodology, the MEGA search methodology evaluates structures in parallel. Once the minimized isomers have been selected, they will be subjected to either crossover or mutation operations. As part of the tournament selection method,^52^ minimized cluster pairs are selected for crossover procedures by using a fitness criterion. Based on the cut-and-splice method developed by Deaven and Ho,^64^ offspring clusters are generated. MEGA applies mutation operations randomly on the other minimized clusters using four operators: “rotate”, “move”, “twist”, and “atom inversion”. The population size was set to 12 structures, and the mutation rate (*P*_mut_) was fixed at 20%, with mutation types chosen randomly from four available operators: atom displacement, rigid rotation, cluster twist, and atom inversion. The remaining 80% of new structures were generated *via* crossover operations using a variant of the cut-and-splice method, mentioned above, where fragments from parent clusters were combined to produce offspring. Fitness functions were dynamically scaled based on the normalized energy difference relative to the best and worst members of the population, ensuring robust selection. Pool diversity was maintained by using geometric similarity checks based on sorted interatomic distance lists, with a threshold of 5% to differentiate structures. Convergence was monitored by ensuring that the five lowest-energy structures remained unchanged for at least 50 iterations. These parameter choices and algorithms were optimized for computational efficiency and have been validated against benchmark systems, as detailed in the ref. 58 and 59. It is worth noting that for each cluster size, more than 500 geometries were generated initially using MEGA. Subsequently, the process was refined using partially filled pools and iterative screening. Fewer than 300 structures were evaluated for each cluster size during the independent GA cycles for the systems. These steps ensured that the final lowest-energy structures for the clusters were determined after significant computational optimization. Refer to ref. 58 and 59 in the references for further details on the MEGA code.

Gamma-point, spin-polarized and periodic Density Functional Theory (DFT) calculations are conducted using the revised Perdew–Burke–Ernzerhof (rev-PBE) parameterization for the exchange–correlation functional. Rev-PBE functional was selected for this study because of its well-documented performance in describing metallic systems and dispersion interactions, both of which are critical for accurately capturing the structural and energetic properties of Cu–In nanoclusters. Previous studies have shown that rev-PBE provides reliable predictions for metallic bonding and cluster stability, particularly in systems involving transition metals.^65,66^ In addition, the computational efficiency of rev-PBE makes it well-suited for global optimization studies using approaches such as the Mexican Enhanced Genetic Algorithm (MEGA), where large numbers of structures are evaluated.^59^ This balance of computational efficiency and accuracy was critical for our study. Spin polarization was incorporated to accurately capture magnetic behaviors, especially for odd-electron clusters. This approach was vital for determining ground-state spin configurations and understanding magnetic contributions to stability. Projected-augmented wave (PAW) pseudopotentials are used to replace the core electrons, ensuring efficient and accurate representation of core–valence interactions.^67,68^ Plane-wave basis sets (with a kinetic energy cutoff of 450 eV)^69^ are used to describe the valence electrons: 11 electrons of Cu (3d^10^ 4s^1^) and 3 electrons of In (5s^2^ 5p^1^), provided a balance between computational cost and precision, capturing the critical electronic features of Cu and In clusters. The relaxation of the atomic positions in the supercell occurred until the forces were smaller than 0.01 eV Å^−1^. Methfessel–Paxton smearing, with a sigma value of 0.01 eV was used to improve metallic convergence.^70^ Through frequency analysis of the DFT energy surface, it was verified that the structures obtained are indeed true minima. Electronic structure simulations are conducted using the Heyd–Scuseria–Ernzerhof (HSE06) hybrid function;^71^ to ensure more accurate electronic properties. The HSE06 hybrid functional was chosen for electronic property calculations due to its well-established ability to provide a more accurate description of band gaps and electronic states compared to generalized gradient approximation (GGA) functionals like rev-PBE. HSE06's incorporation of a portion of exact exchange effectively corrects the underestimation of band gaps typically observed in GGA functionals, making it particularly suited for studying the electronic properties of Cu–In clusters. Our choice of combining rev-PBE for structural optimizations and HSE06 for electronic property calculations is based on a balance of computational efficiency and accuracy. Rev-PBE was selected for geometry optimization due to its reliability in describing metallic bonding and dispersion interactions, while HSE06 was employed to achieve improved accuracy in the electronic structure. This approach has been validated in previous studies involving metal clusters and nanoalloys.^68,71^

We have conducted additional convergence tests for total energy, forces, and key structural parameters (*e.g.*, bond lengths) as a function of the plane-wave cutoff energy. These results confirm that a cutoff energy of 450 eV is sufficient to achieve convergence within a tolerance of 0.01 eV per atom for total energy and negligible deviations in forces and bond lengths. Similarly, we have verified that a Gamma-point-only *k*-point sampling is appropriate for these small, gas-phase clusters, as the supercell size (15 × 15 × 15 Å^3^) effectively minimizes spurious periodic interactions. We have also tested larger supercells (*e.g.*, 18 × 18 × 18 Å^3^ and 20 × 20 × 20 Å^3^) and observed negligible differences in total energy (<0.01 eV per atom), binding energy, and electronic structure, confirming that the chosen supercell size effectively eliminates periodic interactions. Our choice of combining rev-PBE for structural optimizations and HSE06 for electronic property calculations is based on a balance of computational efficiency and accuracy. Rev-PBE was selected for geometry optimization due to its reliability in describing metallic bonding and dispersion interactions, while HSE06 was employed to achieve improved accuracy in the electronic structure. This approach has been validated in previous studies involving metal clusters and nanoalloys.

All calculations in this study employed Projector Augmented-Wave (PAW) pseudopotentials as implemented in the Vienna *Ab initio* Simulation Package (VASP). The default pseudopotentials from the 2018 VASP database were used. For copper (Cu), the standard potential was selected, which includes the 3d^10^ 4s^1^ 3d electrons as valence states, while the semicore 3p states were excluded. For indium (In), the standard potential was utilized, which includes the 4d^10^ 5s^2^ 5p^1^ electrons as valence states, thereby accounting for the semicore 4d states. These choices provide a balance between computational efficiency and accuracy, as the inclusion of Cu 3p semicore states is generally unnecessary for the systems studied, while the In 4d states are closer to the valence levels and can influence bonding and electronic structure. During geometry optimizations, the convergence criteria were carefully set to ensure reliable results. The force convergence threshold was set to 0.01 eV Å^−1^, as specified by the tag EDIFFG = −0.01 in the INCAR input file. This ensures that the forces on each atom are below this threshold, indicating that the system has reached equilibrium with minimal atomic displacements. Additionally, the energy convergence threshold for the self-consistent field (SCF) calculations was set to 10^−5^ eV (default value in VASP), ensuring that the total energy change between successive electronic steps is below this limit. These criteria guarantee both structural and electronic accuracy during the simulations.

Zero-point energy (ZPE) corrections were not explicitly applied to the calculated binding energies or total energies of the clusters. While it is acknowledged that ZPE contributions can influence the accuracy of energy differences, particularly for small clusters, prior studies have shown that these corrections often result in only minor shifts in relative energy rankings for clusters of the sizes examined here. Therefore, the primary conclusions regarding structural stability and energy trends are not expected to be significantly impacted by the exclusion of ZPE corrections. However, we note that in a similar study on AuCu clusters,^31^ we have explicitly calculated ZPE corrections at the harmonic level and applied to the binding energies. This study also confirmed that ZPE contributions had a limited effect on the energy differences between clusters but provided a refined understanding of the adsorption energetics. Based on this, we conclude that while ZPE corrections could add further precision, their exclusion in the present study is unlikely to alter the primary conclusions.

The Visualization for Electronic and Structural Analysis (VESTA)^72^ is used to envisage the geometries of the nanoparticles, while SUMO^73^ is used for plotting electronic structure graphs. Point groups are generated using VASPKIT.^74^ In order to investigate the nature of interactions within clusters, a number of physical properties on a per-atom basis are examined by employing the Quantum Theory of Atoms in Molecules (QTAIM) using AIM2000 program,^75^ the corresponding wave functions are generated at the B3lyp/WTBS level of theory, as this combination provides detailed insights into bonding characteristics and electronic distributions, complementing the results from the periodic DFT calculations.

### 2.2. Energetics analysis

The average binding energy per atom (*E*_b_), that is related to the stability of nanoalloys, can be computed as follows:^51^1
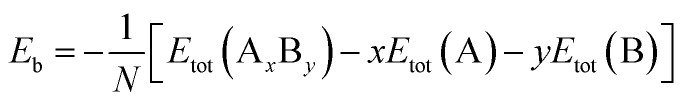
where *N* is the total number of atoms (*N* = *x* + *y*). *x* and *y* are the numbers of atoms A (Cu) and B (In), respectively. *E*_tot_(A), *E*_tot_(B) and *E*_tot_(A_*x*_B_*y*_) are the total energies of pure atoms Cu and In and the bimetallic CuIn nanoparticles, respectively.

The comparison of the stability of nanoalloys with different compositions requires the use of a mixing (or excess) energy term (*Δ*) according to the equation below:^51^2
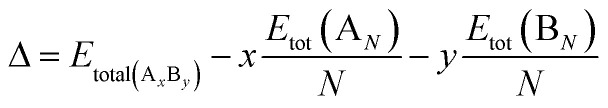


The total energy of a nanoalloy is denoted by *E*_tot(A_*x*_B_*y*_)_. *E*_tot_(A_*N*_) and *E*_tot_(B_*N*_) are the energies of the pure Cu and In nanoparticles with the same size as Cu_*x*_In_*y*_, respectively. Generally, this excess energy is an unbiased quantity, defined as zero for the global minima of the pure clusters. A negative mixing energy (*Δ*) implies a decrease in energy after mixing, consequently a favorable mixing; while a positive value indicates a de-mixing tendency.

The second-order difference of the binding energy (Δ_2_*E*) of pure Cu and In and their mono-substituted clusters is a sensitive quantity, that reflects the relative stability of nanoalloy of size *N* with respect to its neighbors (*N* + 1 and *N* − 1 sizes), and it can be computed by:^50^

3 Δ_2_*E* = *E*(A_*N*+1_) + *E*(A_*N*−1_) − 2*E*(A_*N*_)]

The ionization energies (*I*) and electron affinities (*A*) for nanoclusters were calculated using Koopman's approximation:^76^

4 *I* = −*E*_Homo_

5 *A* = −*E*_LUMO_

Following this, *I* and *A* were subsequently used to compute the conceptual DFT-based descriptors: electronegativity (*χ*), global hardness (*η*), molecular softness (*S*), and electrophilicity index (*ω*), which are given by the formula:6
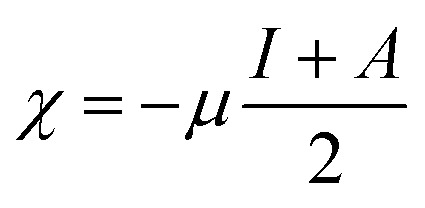
where (*μ*) represents the chemical potential of a given system.7
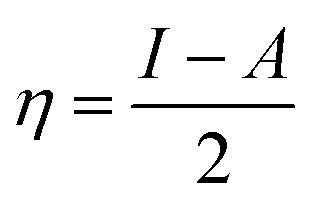
8
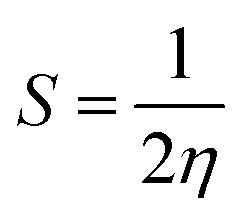
9
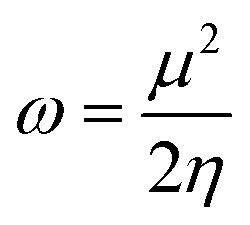


## 3. Results and discussion

The spin states of all atoms, dimers, and the lowest energy monosubstituted clusters produced by MEGA were optimized using VASP. It was observed that all pure Cu species displayed the lowest spin states achievable: singlet states for clusters with an even number of electrons (even-*N* clusters) and doublet states for clusters with an odd number of electrons (odd-*N* clusters). In contrast, the spin states for Cu-doped, In-doped, and In species exhibited non-monotonic trends. Table S1 in the ESI† shows the optimal spin state for each species.

### 3.1. Structural analysis

#### 3.1.1. Pure clusters

**Fig. 1:**
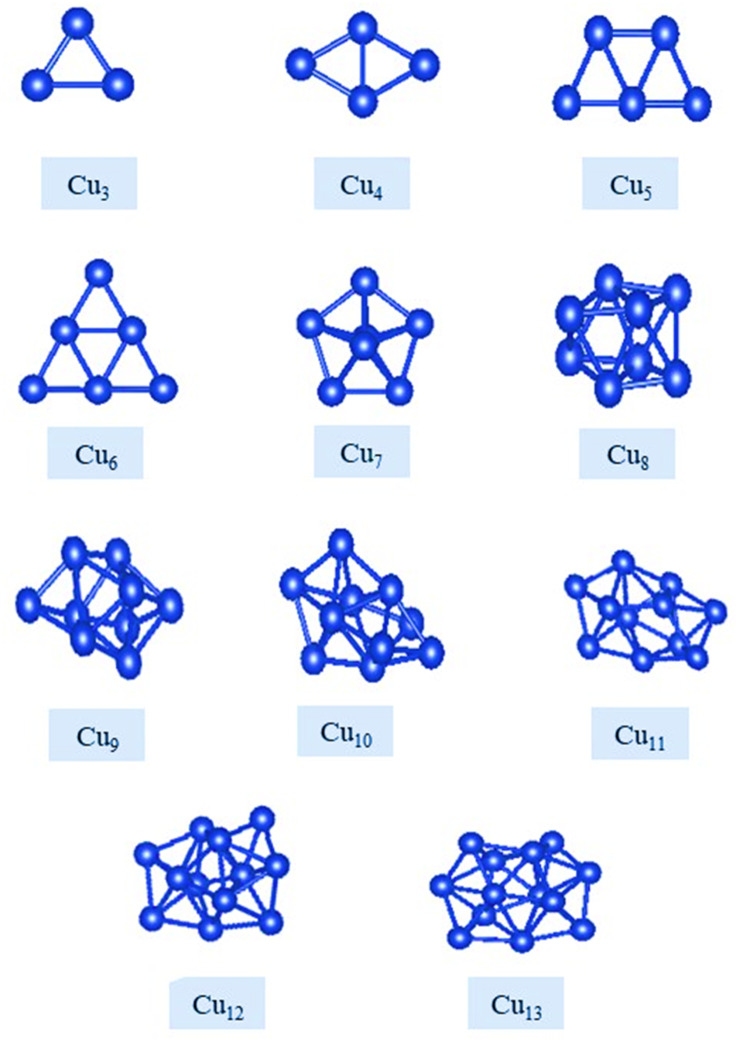
Putative global minimum structures for Cu nanoalloys, *N* = 3–13.

The putative global minima for pure Cu nanoalloys 3 ≤ *N* ≤ 13, are shown in Fig. 1, their energies, point groups, and their *XYZ* coordinates are listed in ESI.† The GMs of the free Cu clusters (*N* = 2 to 6) are found to have 2D configurations. In this way, the 5d–6s energy separation is decreased, which strengthens the s–d hybridization.^77^ Looking at Fig. 1, Cu_3_ cluster has a equilateral triangular geometry with an angle of 67.11°, in agreement with the literature.^31^ According to the results obtained for Cu_*N*_ (*N* = 4–7), the MEGA-generated geometries are consistent with findings of previous studies.^78–81^

**Fig. 2:**
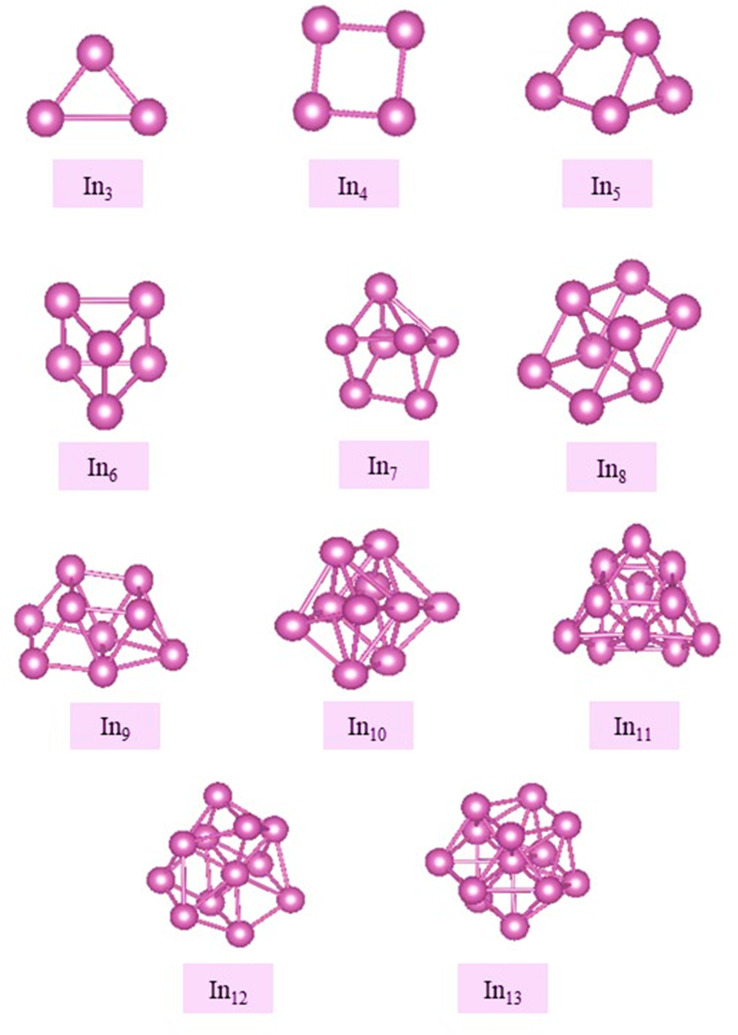
Putative global minimum structures for In nanoalloys, *N* = 3–13.

Indium clusters are found to be a 2D structure for *N* = 2–4, where *N* = 3 takes a triangle shape and *N* = 4 takes a square shape (see Fig. 2). For sizes *N* = 4–13, indium clusters are found to be 3D structures. The GM located for In clusters with sizes (*N* = 2–10) agree with the findings of previous studies^82^ although their suggested geometries were not globally optimized. The bond distance is found to be 2.22 Å for Cu dimer, and 3.06 Å for In dimer. In comparison with results of previous experimental and theoretical studies, these dimensions are in good agreement,^83–86^ as listed in Table S1 in the ESI.† The results refer that the bond length of Cu–In dimer is 2.534 Å, therefore, the properties of Cu_*n*_In_*m*_ clusters are supposed to lie between those of Cu and In clusters.

#### 3.1.2. Single-atom nanoalloys

**Fig. 3:**
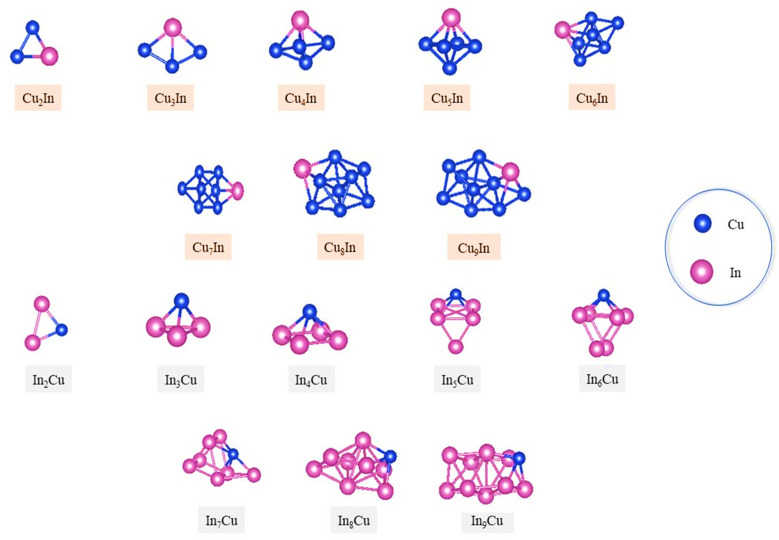
Putative global minimum structures for copper–indium nanoalloys, *N* = 3–10.

Fig. 3 shows the putative global minima for (3 ≤ *N* ≤ 10) copper–indium nanoalloys and their *XYZ* coordinates are listed in the ESI.† Their energies, average bond distances, point groups and spin states are listed in Tables S2 and S3 in the ESI.†

As a result of “homotops” (*i.e.* isomers that have the same structure, total number of atoms, and composition but are different in their chemical ordering) in bimetallic clusters, finding the global minima becomes increasingly difficult.^87^ In In-doped Cu clusters Cu_*n*_In_1_ (where *n* ranges from 2 to 5), the planar structural configuration typical of pure Cu clusters is disrupted. This finding contrasts with previous research,^38,80^ which suggested that the presence of the In atom does not dramatically affect the nature of Cu clusters. Specifically, while earlier studies predicted that doped clusters would maintain similar planar configurations to their pure Cu counterparts, our observations indicate while pure Cu clusters transition from planar to three-dimensional structures at *n* = 7, In-doped Cu clusters show this transition at *n* = 5. Similarly, to Cu_3_ and In_3_, Cu_2_In and In_2_Cu geometries are all found to be bent triangle structures, this can be attributed to the closeness in bond lengths between Cu–Cu (∼2.3 Å) and Cu–In (∼2.645 Å), as shown in Table S3 in the ESI.† For most In-doped Cu clusters Cu_*n*_In_1_ (with *n* ranging from 2 to 9), the global minima (GMs) differ from those of their pure Cu counterparts of the same size (Cu_*n*+1_), except for Cu_2_In_1_ and Cu_3_In_1_, where the presence of the doped In atom does not alter their structural configurations. These dopant-induced structural modifications have been anticipated for larger Cu–In clusters, as predicted in previous studies.^38,57,88^ The global minima obtained by our MEGA-DFT approach for sizes 2 ≤ *N* ≤ 5 are in fair agreement with those found by previous published studies,^80,82^ despite the fact that the structures were not globally optimized. According to our findings, it is suggested that indium atoms tend to occupy low-connectivity sites at the surfaces and edges, thereby reducing the number of bonds, one of the factors that may delay the formation of 3D configurations for the greater sizes. We can conclude, from examining the distribution of Cu and In atoms over the entire clusters, that there is a clear tendency for Cu atoms to be located at interior sites. This has previously been reported for larger clusters.^38^ This can be attributed to the bigger atomic radius of indium relative to Cu (In = 1.66 Å, Cu = 1.28 Å (ref. 78)) as well as the lower surface energy and the higher electronegativity of In atom also play a crucial role in this context.^89^

To further investigate the structural and electronic properties of the clusters, we calculated the coordination numbers of Cu and In atoms in each cluster. The coordination number of an atom was defined as the number of nearest neighbors within a cutoff distance determined from the sum of the covalent radii of Cu and In, plus a 0.5 Å tolerance to account for bond length variations. In clusters with a higher proportion of Cu atoms, the average coordination number of Cu was found to be 4.2, indicating a compact metallic core structure. Conversely, In atoms in these clusters had a lower average coordination number of 3.1, suggesting that they preferentially occupy surface or peripheral sites. For clusters with a more balanced Cu : In ratio, both Cu and In atoms exhibited intermediate coordination numbers, with averages of 3.8 and 3.5, respectively, reflecting a more homogeneous distribution of the two elements. In clusters dominated by In, the coordination number of Cu dropped to 2.5, indicating segregation tendencies where Cu atoms favor positions surrounded by In atoms. These coordination trends provide valuable insights into the mixing behavior of Cu and In. The higher coordination of Cu atoms highlights their tendency to form a compact core, while the lower coordination of In atoms suggests their preference for surface positions, consistent with their larger atomic size and lower cohesive energy. This segregation behavior and its impact on electronic structure could play a critical role in determining the catalytic activity of these clusters.

To complement the discussion of average bond lengths, we performed a statistical analysis of the bond length distributions for Cu–Cu, Cu–In, and In–In interactions within the clusters. The standard deviations and ranges of bond lengths were calculated to capture the structural diversity. For Cu–Cu bonds, the lengths ranged from 2.38 Å to 2.55 Å, with a standard deviation of 0.06 Å, reflecting the relatively uniform bonding environment of Cu atoms. Cu–In bonds exhibited a broader range of 2.50 Å to 2.78 Å and a higher standard deviation of 0.09 Å, indicating greater variability in the bonding interactions due to size mismatches between Cu and In atoms. In contrast, In–In bonds showed the widest range of 2.80 Å to 3.12 Å with a standard deviation of 0.11 Å, consistent with the weaker cohesive energy and larger atomic size of indium.

These variations in bond lengths are correlated with the stability trends observed for different cluster compositions. The narrow distribution of Cu–Cu bond lengths suggests a strong preference for uniform metallic bonding, whereas the broader distributions for Cu–In and In–In bonds reflect the strain and geometric adjustments required to accommodate the heteroatomic interactions. These insights provide a more nuanced understanding of the structural diversity within the clusters, which could influence their catalytic properties by modulating electronic structure and active site accessibility.

#### 3.1.3. Comparison with previous work

To provide a comprehensive context, we compare our results with previous studies, discussing both Cu-rich (Cu_*x*_In) and In-rich (CuIn_*n*_) clusters. The obtained lowest-energy structures and their relative stabilities were validated against prior theoretical and experimental findings, demonstrating overall agreement with minor deviations due to methodological differences. For small Cu-rich clusters, such as Cu_3_In and Cu_4_In, our results show that Cu_3_In adopts a planar triangular geometry, while Cu_4_In forms a distorted tetrahedron with indium preferentially occupying a vertex site. These findings are in agreement with the studies of ref. 46, 79 and 90, although variations in relative energies (within 0.05 eV) highlight the sensitivity of density functional theory (DFT) to the choice of exchange–correlation functional. Larger Cu-rich clusters (*e.g.*, Cu_6_In and Cu_8_In) exhibit compact three-dimensional (3D) configurations, with indium preferentially positioned at capping or substitutional sites, a trend also reported by ref. 79.

Indium-rich clusters exhibit distinct structural motifs, diverging from Cu-rich clusters in both dimensionality and bonding preferences. CuIn_2_ is found to adopt a bent structure, while CuIn_3_ forms a pyramidal arrangement. These results align with the work of ref. 86, 90 and 91, which observed a strong preference for indium atoms to occupy low-connectivity sites due to their larger atomic radius and lower cohesive energy. For CuIn_4_ and CuIn_6_, the clusters transition into planar and quasi-hexagonal geometries, respectively, with copper atoms playing a secondary role in structural stabilization. Notably, the energetic ordering of these clusters varies depending on the choice of functional; for instance, the Cu_4_In and Cu_6_In clusters show slight reordering when hybrid functionals such as HSE06 are employed, compared to generalized gradient approximation (GGA) functionals.

Looking more closely at the lowest-energy isomers for both Cu_*m*_In_1_ and Cu_1_In_*n*_ clusters, along with their relative energies and comparisons with literature values, we can observe the discrepancies primarily stem from differences in computational settings, such as basis sets, functional choices, and the inclusion (or omission) of dispersion corrections. Unlike previous studies, which often relied on local optimization techniques, our approach integrates a global optimization strategy (MEGA-DFT), ensuring an exhaustive search for the most stable configurations. This methodological refinement enables a more robust prediction of cluster geometries while accounting for computational uncertainties and functional-dependent energy variations.

Our results for the structural preferences and dimensionality of pure Cu and In clusters align well with earlier studies. Specifically, we observed that pure Cu clusters transition from planar to three-dimensional geometries at *n* = 7, consistent with prior findings.^78–81^ Similarly, pure In clusters transition to 3D structures at *n* = 5, which is in agreement with reports by Zhang *et al.*^65^ and other theoretical studies, though those studies lacked global optimization.

For Cu–In clusters, our observation that indium doping accelerates the structural transition to 3D at *n* = 5 is consistent with previous computational predictions.^38,57,88^ However, our global optimization approach revealed unique low-energy configurations for doped clusters that deviate slightly from earlier reported planar structures. This discrepancy highlights the importance of using advanced global optimization methods like MEGA.

The close agreement between our calculated bond lengths (*e.g.*, Cu–In at 2.645 Å) and experimental/theoretical values reported in prior works^83–86^ further validates our results. However, our results refine earlier structural predictions, showing a preference for indium atoms to occupy low-coordination sites, contrary to assumptions in studies that lacked explicit global optimization.

To address the concern regarding functional dependency, we listed several low-energy configurations and their relative energies in Table S4 of the ESI.† This allows for a direct comparison and ensures that potential functional biases are acknowledged. Our results indicate that while the exact energy rankings may vary slightly with the functional used, the structural trends remain consistent across different methodologies.

### 3.2. Energetic stability

The energetic characteristics of atomic site preferences, structural behavior, and their nanocluster stability can be assessed by computing several energetic criteria, such as: binding energy (*E*_b_), mixing energy (*Δ*), and second difference energy (Δ_2_*E*), eqn (1)–(3). Listed in Table S4 in the ESI† are the values of these energies for Cu, In, and bimetallic systems.

**Fig. 4:**
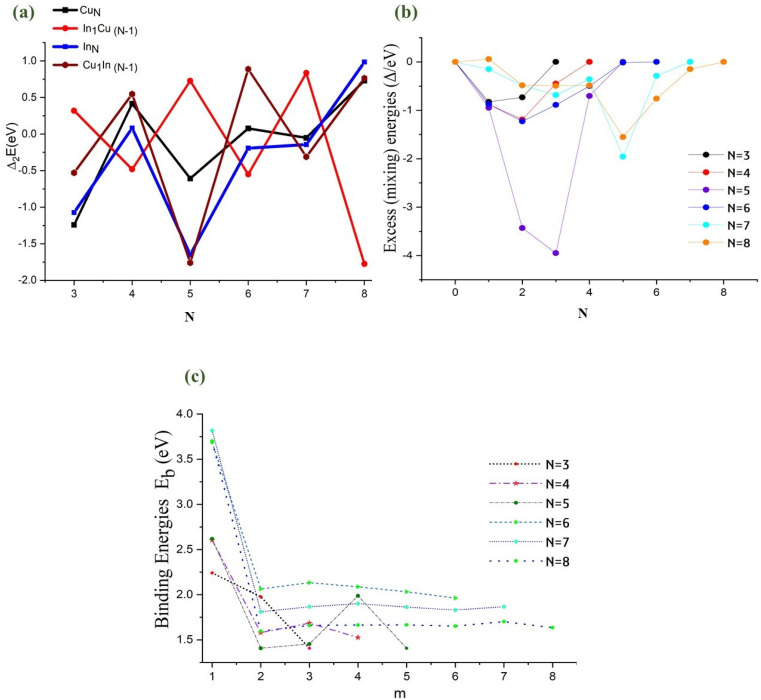
(a) Plot of second difference in energy Δ_2_*E* as a function of total number of atoms (*N*) for pure and mono-substituted copper–indium clusters. (b) Plot of excess (mixing) energy *Δ* against the number of In atoms for copper–indium clusters, with *N* = 3–8. (c) Plot of binding energies (*E*_b_) against the number of In atoms (*m*) for copper–indium clusters, with *N* = 3–8.

Using the second difference in energy (Δ_2_*E*), we examine the stability of pure clusters in comparison with their singly doped counterparts. Eqn (3) allows us to compare the stability of clusters with different numbers of atoms by considering their sizes and total number of atoms (relative to neighboring clusters). It provides a quantitative measure of how stable a cluster is relative to its neighboring clusters, taking into account the crucial factors of size and type of atoms. The second difference in energy for pure monometallic Cu and In clusters, and their mono-substituted clusters as a function of cluster size, are plotted in Fig. 4(a). Positive peaks in the second difference in energy plot indicate the high relative stability of clusters compared to their nearest neighbor clusters with one atom less or more. Among the monometallic nanoalloys, it can be observed that Cu_8_ (3D) and In_8_ (3D) clusters exhibit the strongest relative stabilities. The positive peaks observed for the even-*N* clusters of Cu are indicative of the relative stability of their sizes, whereas the reverse is observed for the odd-*N* clusters (negative troughs). It has been proposed that this even–odd behavior is explained by an electronic shell model,^28,31,92,93^ in which even-electron clusters (*e.g.* Cu clusters) have a higher stability due to the contribution of one 4s electron per atom to the delocalized cluster bonding, which leads to enhanced stability.^28,31,93^

A comparison between pure Cu_*N*_ clusters and other systems reveals an interesting trend in their relative stabilities. In_*N*_ and Cu_1_In_(*N*−1)_ clusters exhibit the same even–odd fluctuation seen in Cu_*N*_ clusters, but with notable differences in the intensity of their positive peaks and negative troughs. In contrast, In-doped Cu clusters display a reversed stability pattern, showing an odd–even trend of stable-unstable. For pure clusters, the highest stability, indicated by positive peaks in Δ_2_*E*, occurs at *N* = 8. In the case of In-doped Cu clusters and Cu-doped In clusters, the highest stability is observed at *N* = 7 and *N* = 6, respectively.

The stability of Cu–In clusters is heavily influenced by the effects of mixing. To examine these effects, particularly those induced by doping, we calculated the mixing (excess) energy (*Δ*). Fig. 4(b) presents a graphical analysis of the mixing energy for Cu–In clusters with *N* = 3–8 atoms. Negative *Δ* values indicate a strong tendency for mixing, whereas positive values suggest an unfavorable mixing tendency. The results reveal that all mixed clusters, with the exception of the bimetallic nanoclusters Cu_7_In_1_ and In_5_Cu_3_, exhibit favorable negative mixing energy values, with these two exceptions displaying a slight unfavorable positive mixing energy of 0.05 eV. For nanoclusters with a constant atomic count, compositional variations exert a more significant effect on overall stability than geometric changes, as reflected in the mixing energy values. The analysis further shows that the configuration with the highest stability is achieved when the In proportion is approximately 50%. Overall, the negative mixing trend for CuIn clusters ranges from 0.0 to around −3.95 eV, with the lowest *Δ* value – indicating the greatest propensity for mixing – observed in Cu_2_In_3_ (−3.94 eV), followed by Cu_3_In_2_ (−3.43 eV). These findings suggest a preference for In atoms to occupy low-coordination sites, such as edges, a trend that may lead to surface segregation. This suggests that optimal composition and positioning can maximize stability in bimetallic clusters, and our results are consistent with previously predicted structures for larger Cu–In clusters.^38^

The relative stabilities of bimetallic clusters can be also assessed by examining their atomic arrangements through the binding energy per atom (*E*_b_). A graphical representation of the binding energies for Cu–In nanoalloys is provided in Fig. 4(c). In general, an increase in binding energy signifies enhanced stability. As observed in this study, larger clusters exhibit higher binding energies, which can be attributed to the increased average number of metal–metal bonds, a trend consistent with other systems.^94,95^ In comparison, nanoclusters composed of pure Cu or Cu-rich compositions demonstrate greater stability than those composed of pure In or In-rich compositions, a pattern also observed in other systems.^28,31,58^ Comparatively, nanoclusters with pure Cu or Cu-rich compositions exhibit greater stability than those composed of pure In or In-rich compositions. This is evidenced by their higher positive binding energy values, which imply stronger metal–metal interactions and enhanced structural stability. These results highlight the critical role of composition in influencing the stability of bimetallic nanoclusters, with Cu-rich clusters demonstrating superior binding energy for clusters of the same size. Consequently, the Cu–In ratio significantly contributes to the overall stability of the cluster structure.

To gain deeper insights into the stability of bimetallic In–Cu clusters, we decomposed the total energy into contributions from Cu–Cu, Cu–In, and In–In interactions. This analysis was performed using pairwise energy decomposition techniques based on the computed electronic structure. Our results reveal that Cu–In interactions consistently exhibit the most negative energy contributions, indicating a strong energetic preference for heteroatomic bonding. In contrast, Cu–Cu and In–In interactions (as will be discussed in Section 3.3.2) are comparatively weaker, with In–In interactions being the least favorable due to the lower cohesive energy of indium relative to copper. For the In_4_Cu_1_ cluster, the high stability is attributed to the maximization of Cu–In interactions, which stabilize the cluster by balancing the strain induced by size mismatches between Cu and In atoms. Clusters with compositions closer to a 1 : 1 Cu : In ratio exhibit enhanced mixing tendencies, as evidenced by the significant negative values of mixing energy, making them particularly favorable for catalytic applications. These findings align with previous studies, which suggest that heteroatomic bonding in bimetallic systems can modify electronic properties and enhance catalytic activity. The decomposition analysis further suggests that fine-tuning the Cu : In ratio can optimize the stability and reactivity of the clusters for specific catalytic processes, such as CO_2_ reduction or hydrogen evolution.

The interaction energies and total energies for Cu and In clusters were computed using plane-wave basis sets within the VASP framework. Plane-wave basis sets, as opposed to localized Gaussian-type orbitals, are designed to mitigate Basis-Set Superposition Error (BSSE) due to their inherent completeness in describing periodic systems. Consequently, explicit BSSE corrections, such as the counterpoise method, were not applied. However, we acknowledge that residual BSSE effects could still influence the interaction energies of non-periodic systems, such as small dimers or clusters. To ensure reliability, we employed a high kinetic energy cutoff (450 eV) and verified the convergence of our calculations, which aligns with best practices for minimizing such effects. Moreover, the consistency of our calculated bond lengths and energetics with experimental and theoretical values reported in the literature lends further confidence to the accuracy of the results (see Section 3.1.2).

The convex hull (Fig. S7†) highlights the stability transitions across compositions. Energies gradually decrease as the composition shifts from pure Cu to mixed clusters, demonstrating favorable mixing. However, energy increases again as the composition shifts towards pure In. Smaller clusters such as Cu_2_, Cu_3_, and Cu_4_ generally show higher stability due to increased binding energy per atom, especially when mixed with In. Pure In clusters (*e.g.*, In_2_, In_3_, In_4_) also display stable configurations, indicating the intrinsic stability of In at certain cluster sizes. Clusters lying on the convex hull are thermodynamically stable, as they represent the lowest energy configurations for a given composition. For example, pure Cu_2_ (In fraction = 0.0), Cu_1_In_1_ (In fraction = 0.5), and pure In_2_ (In fraction = 1.0) are stable configurations since they fall on the convex hull. Points off the convex hull represent less stable or metastable clusters, which may decompose into mixtures of clusters represented by the hull vertices. For instance, In_1_Cu_2_ and In_2_Cu_1_ have slightly higher energies compared to their neighboring stable configurations, suggesting reduced stability. The minimum energy configurations tend to cluster around compositions where the proportion of Cu and In atoms are either pure or equimolar (*e.g.*, Cu_1_In_1_ with In fraction = 0.5). This indicates that mixing between Cu and In is energetically favorable in specific ratios, particularly at an equal Cu : In ratio. The diagram reveals a strong preference for certain mixed compositions, suggesting that alloying Cu and In can stabilize the clusters under certain conditions. Clusters with compositions far from the convex hull are less likely to form or may undergo decomposition into more stable configurations. The data provides insights into the energetically optimal cluster compositions and can guide the synthesis of stable Cu–In nanoalloys for targeted applications, such as catalysis.

### 3.3. Physico-chemical properties

#### 3.3.1. Electronic properties

**Fig. 5:**
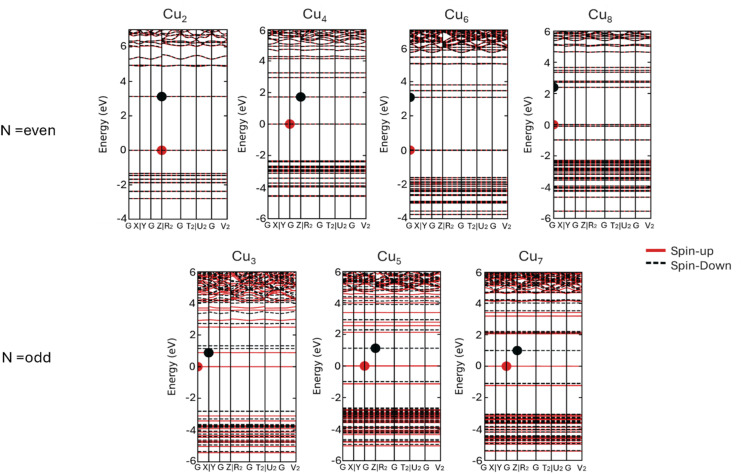
Spin projected band structures of monometallic Cu_*N*_ at HSE06 level.

**Fig. 6:**
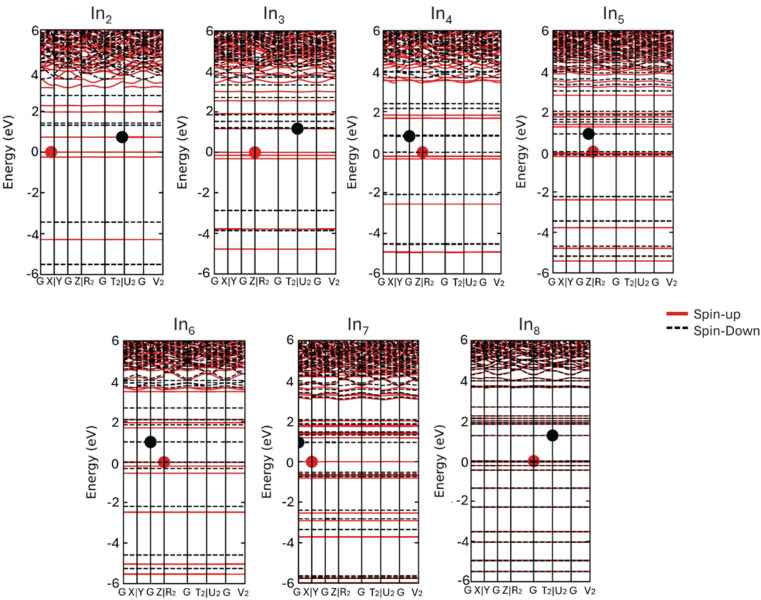
Spin projected band structures of monometallic In_*N*_ at HSE06 level.

As a means of understanding the electronic properties of these clusters, we carried out band structure simulations. Fig. 5 illustrates the spin-projected band structures of all Cu_*N*_ (*N* = 2–8) clusters, which proves their magnetic behaviors. As shown in Fig. 5, a Cu cluster containing an odd number of electrons exhibits spin polarization. On the other hand, Cu_*N*_ clusters containing an even number of electrons show symmetric spin-up and spin-down bands, which is indicative of the non-magnetic nature of these clusters. The findings of this study are in good agreement with those reported in previous publication.^96^ Interestingly, all monometallic In_*N*_ (*N* = 2–8) clusters show a characteristic magnetic behavior except for the cluster In_8_ (see Fig. 6). Additionally, the electronic structure analysis reveals that Cu clusters (except Cu_4_) have direct gaps; owing to the conduction band minimum (CBM) and the valence band maximum (VBM) being at the same point in the Brillouin zone. Moreover, all magnetic Cu clusters and all In_*N*_ clusters have indirect gaps. The calculated band gaps at the HSE06 level are listed in Table S5.†

**Fig. 7:**
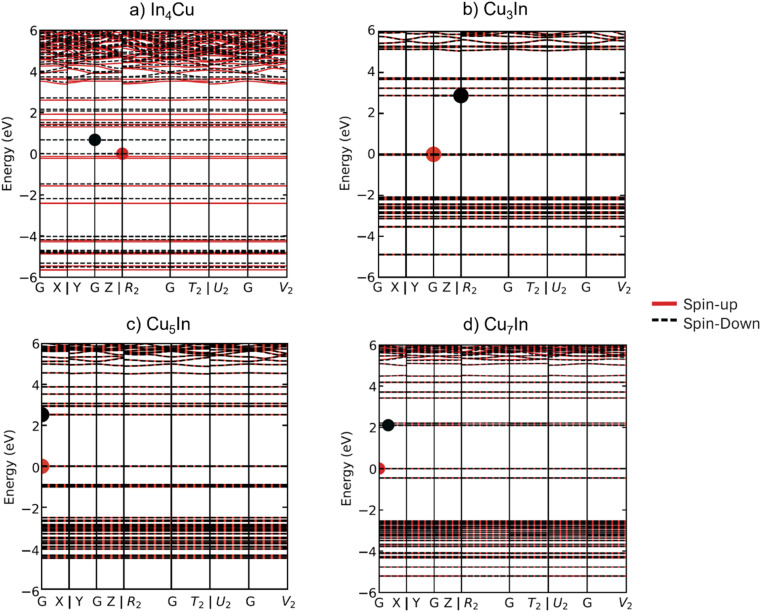
Spin projected band structures of selected bimetallic clusters at HSE06 level for (a) In_4_Cu, (b) Cu_3_In, (c) Cu_5_In, and (d) Cu_7_In.

Moving on to bimetallic nanoclusters, we have plotted the spin-projected bands of four different nanoclusters in order to compare their electronic properties, In_4_Cu, Cu_3_In, Cu_5_In, and Cu_7_In (see Fig. 7). Starting by first examining the band structure of In_4_Cu (Fig. 7(a)). It can be observed that the In_4_Cu band structure exhibits magnetic behavior similar to that of monometallic In_5_. In these two band plots, it is clear that the spin polarization is observed predominantly around the Fermi level. The magnetic properties of the clusters were investigated by calculating their total magnetic moments and analyzing the spin density distributions. For the In_4_Cu_1_ cluster, a total magnetic moment of 1.12 *μ*_B_ was obtained, primarily arising from the unpaired electrons localized on the Cu atom and partially delocalized over the adjacent In atoms. Spin density plots, provided in the ESI material,† visualize the distribution of spin density within the clusters, confirming that the magnetic behavior is strongly influenced by the composition and arrangement of the cluster atoms. Specifically, clusters containing Cu exhibit localized spin contributions due to the partially filled 3d orbitals of Cu, while the In atoms contribute to the overall spin delocalization.

The magnetic properties of these clusters could have important implications for catalytic applications. Magnetism can influence the adsorption and activation of reactants by modifying the electronic structure and surface reactivity. For instance, the presence of a magnetic moment could enhance the adsorption of paramagnetic species, potentially altering reaction pathways and activation energies. This interplay between magnetic properties and catalytic behavior warrants further investigation, particularly for clusters with compositions optimized for specific catalytic processes.

To gain a more in-depth understanding of the electronic structure and origin of magnetism of In_4_Cu, we have plotted the partial density of state (PDOS) and total density of state (TDOS) of the In_4_Cu and the In_5_ clusters (see Fig. S1†). It can be seen in the PDOS that the CBM and VBM are dominated by the In atom, indicating that the In atom contributes a significant amount to the total magnetic moment. The most noticeable difference between In_4_Cu and In_5_, is the band gap. Their VBM is located at the *R*_2_ point, but the CBM of In_4_Cu show a small shift toward the *G* point, when compared with the In_5_, leading to reduce band gap to 0.674 eV (see Table S5†).

Based on the HSE06 spin-projected band structure and the PDOS of bimetallic Cu_3_In, Cu_5_In, and Cu_7_In, it is evident that the cluster is non-magnetic, mainly because of the spin-up and spin-down electronic behavior. In addition, PDOS plot (Fig. S1†) indicates that the band gap of monometallic Cu_4_, Cu_6_, and Cu_8_ has changed compared to the band gap for bimetallic Cu_3_In, Cu_5_In, and Cu_7_In, respectively. As a result of the PDOS of Cu_5_In and Cu_7_In, new states, dominated by the In (p) states, were identified at 3 eV and 2 eV, respectively. Consequently, it can be concluded that the presence of In atoms affects the electronic structure of Cu-bimetallic and results in a reduction of the band gap for Cu_5_In and Cu_7_In (2.521 and 2.103, respectively), and an increasing of the band gap for Cu_3_In (see Table S5†).

To provide a deeper interpretation of the electronic structure, we analyzed the distribution of electronic states near the Fermi level and how this distribution varies with cluster size. The projected density of states (PDOS) reveals that, for smaller clusters, states near the Fermi level are predominantly derived from the 3d orbitals of Cu, with minimal contributions from In 5p states. This is consistent with the higher density of states associated with Cu and its tendency to dominate the electronic structure in clusters with fewer atoms. As the cluster size increases and the proportion of In atoms rises, the contribution of In 5p states to the Fermi-level density of states becomes more significant, indicating enhanced hybridization between Cu 3d and In 5p orbitals. This hybridization is crucial for the observed mixing behavior, as it stabilizes the clusters through electronic delocalization. Additionally, clusters with a balanced Cu : In ratio show the most evenly distributed states near the Fermi level, correlating with their enhanced stability and minimal segregation tendencies. For clusters exhibiting magnetic properties, the spin-polarized PDOS shows asymmetry in the spin-up and spin-down states near the Fermi level, primarily localized on Cu atoms. This asymmetry, originating from unpaired 3d electrons, explains the magnetic moments observed in these clusters. The increasing contribution of In 5p states with size suggests a reduction in magnetic moment, as In atoms contribute paired electrons that dilute the spin polarization of Cu. This interplay between electronic state distribution and cluster size provides critical insights into the origin of both stability and magnetism in Cu–In clusters.

The spin-projected density of states (DOS) provides valuable insights into the origin of the magnetic behavior observed in certain clusters. For clusters exhibiting a net magnetic moment, the spin-projected DOS shows a clear asymmetry between the spin-up and spin-down states, particularly near the Fermi level. This asymmetry arises primarily from the unpaired 3d electrons localized on Cu atoms, which dominate the electronic structure in these clusters. The partial density of states (PDOS) further indicates that the spin polarization is most pronounced in the 3d_*xz*_ and 3d_*yz*_ orbitals of Cu, which are partially occupied and contribute significantly to the overall magnetic moment. In clusters with mixed Cu–In compositions, the hybridization between Cu 3d and In 5p orbitals reduces the overall spin polarization by redistributing the electronic states. However, in clusters with a higher proportion of Cu, the spin polarization remains substantial due to the dominant contribution of Cu 3d orbitals. The calculated spin-polarized PDOS also shows that the magnetic moment correlates with the number of unpaired 3d electrons, which decreases as the cluster size increases or as the In content rises. This analysis highlights the interplay between cluster composition, electronic structure, and magnetism. The localization of spin density on Cu atoms and the changes in spin polarization with size and composition are consistent with the observed magnetic moments. These findings suggest that controlling the Cu : In ratio and cluster size could enable the tuning of magnetic properties, which could have implications for catalytic processes involving spin-polarized reactants.

It is possible to determine the chemical stabilities of nanoalloys by analyzing their HOMO–LUMO energy gaps (*Δ*_HL_). Larger values of *Δ*_HL_ indicate high chemical stability of a certain nanoalloy with respect to oxidation (corresponding to a low-lying highest occupied molecular orbital) and reduction (corresponding to a high-lying lowest unoccupied molecular orbital). Table S5 in the ESI† shows values of *Δ*_HL_ as a function of the sizes (*N*) and compositions for all atoms, dimers and clusters. Our analysis reveals that the HOMO–LUMO gap varies significantly with both cluster size and composition. Pure Cu clusters exhibit relatively large gaps, indicating greater stability and lower reactivity, whereas In doping leads to a reduction in *Δ*_HL_, suggesting enhanced electronic flexibility and potential catalytic activity. Notably, Cu–In clusters with a near-equal Cu : In ratio demonstrate moderate *Δ*_HL_ values, balancing stability and reactivity. This electronic tuning effect may enhance catalytic performance in CO_2_ reduction by facilitating charge transfer and stabilizing key intermediates such as *COOH and *HCOO. Additionally, our findings align with previous studies that highlight the role of electronic delocalization in modulating catalytic efficiency in bimetallic systems. Expanding on these insights, future work could explore how HOMO–LUMO trends correlate with experimental catalytic rates, further establishing a direct link between electronic structure and catalytic performance in Cu–In nanoalloys.

#### 3.3.2. Topological analysis

As previously reported^28,31^ clusters with large HOMO–LUMO gaps are expected to exhibit lower reactivity. This relationship can be explained by the concept of hardness (*η*, eqn (7)),^97^ which measures a cluster's resistance to changes in electronegativity or electronic distribution^98^ and is related to transitions between the valence and conduction bands in optical spectroscopy.^78^ The electronegativity (*χ*) values for various compositions are listed in Table S6 of the ESI.† It is observed that Cu and Cu-rich clusters have higher *χ* values (indicating a lower chemical potential) compared to In and In-rich clusters, which accounts for the fact that Cu and Cu-rich clusters retain their electrons more tightly than their In counterparts.

**Fig. 8:**
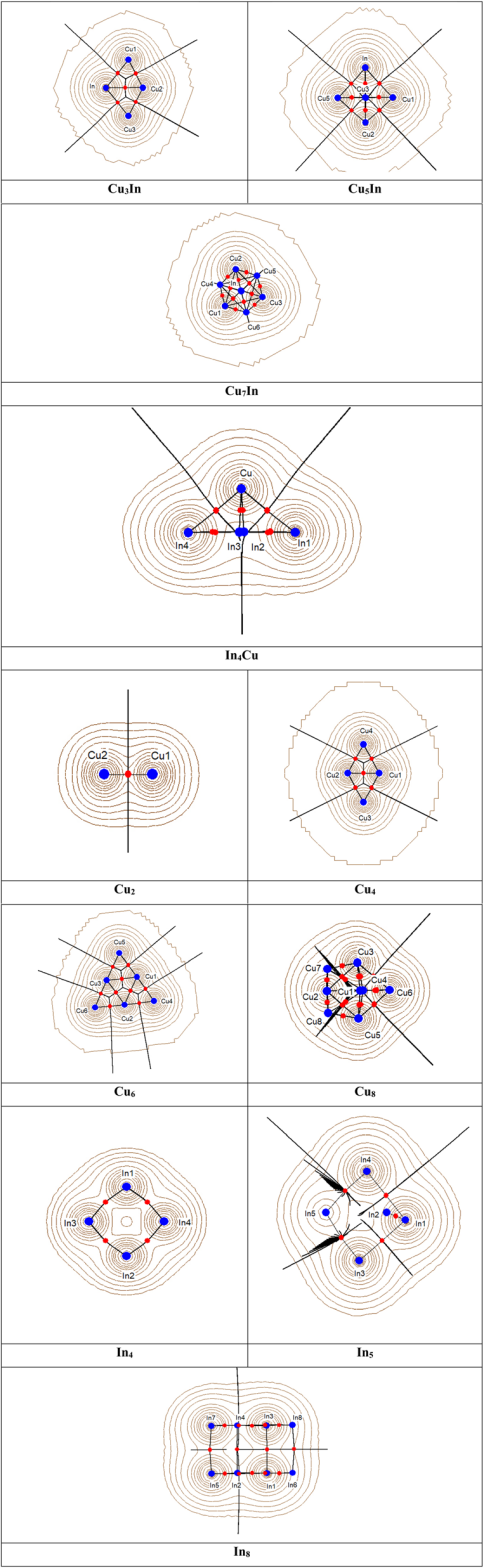
Gradient trajectories mapped on total electron density plots for selected systems from mixed CuIn, and pure Cu and In clusters, showing the atomic basins, bond critical points and ring critical points.

Bader's Quantum Theory of Atoms in Molecules (QTAIM)^99^ is noteworthy for its application of topological information within the electron density, denoted as *ρ*_b_, which is essential for a thorough analysis of the electrical properties and bonding nature in molecular systems. This method has been successfully utilized to examine the interactions in various metal–metal and metal–ligand bonds.^99–101^ In particular, the electronic energy density (*H*_b_), electron density (*ρ*_b_), and its Laplacian (∇^2^*ρ*_b_) offer significant insights into bond interactions.^102^ At the bond critical point, high values of *ρ*_b_, negative values of ∇^2^*ρ*_b_, and the presence of electronic energy density (*H*_b_) indicate a covalent or shared interaction. Conversely, low values of *ρ*_b_, positive ∇^2^*ρ*_b_, and *H*_b_ are characteristic of ionic interactions, similar to closed-shell interactions.^103^ Within the QTAIM framework, the gradient topology of bond critical points and the straight lines connecting interacting atoms constitute a molecular graph.^104^ The molecular graphs for the studied systems (CuIn, InCu, Cu, and In) are depicted in Fig. S4 in the ESI.† These graphs show the positions of all bond critical points and the bond paths that link bonded atoms through these points. For Cu–Cu and In–In bonding interactions, the bond critical points are precisely at the geometrical center of each M–M vector. In contrast, for Cu–In and In–Cu bonding interactions, the bond critical points are located near the center of each In–Cu and Cu–In vector, slightly closer to the Cu atom. The gradient trajectories mapped onto the total electron density plots for all compositions are presented in Fig. 8. This approach also examines properties such as electron density (*ρ*_b_), the Laplacian (∇^2^*ρ*_b_), and electronic energy density (*H*_b_) at the bond critical point, providing crucial information regarding the strengths, characteristics, and types of chemical bonds.

Fig. S5 in ESI† illustrates the Laplacian of the electron density in relevant planes for all clusters, facilitating the analysis of Cu–In, Cu–Cu, and In–In interactions. In this figure, each atom directs a valence shell charge concentration (VSCC) towards a valence shell charge depletion (VSCD) of the Cu and In atoms.

The results of the topological analyses of all bond critical points within the clusters are presented in Table S7 in ESI.† For the Cu–In, In–Cu, Cu–Cu, and In–In interactions, the Laplacian values are positive, ranging from 0.020 to 0.182 eÅ^−3^, while the electron density values are relatively low, between 0.025 and 0.047 eÅ^−3^. The small negative values for the total energy density (*H*_b_), ranging from −0.004 to −0.01 he^−1^, suggest that these interactions are classified as closed-shell metal–metal interactions, increased covalent character. The ratio of potential energy density (*V*_b_) to kinetic energy density (*G*_b_), |*V*_b_|/*G*_b_, serves as an effective tool for characterizing bonds. According to Espinosa and Varadwaj,^105,106^ when |*V*_b_|/*G*_b_ is less than 1 (with ∇^2^*ρ* > 0 and *H*_b_ > 0), it indicates pure “closed-shell interactions”. Conversely, when |*V*_b_|/*G*_b_ exceeds 2 (with ∇^2^*ρ* < 0 and *H*_b_ < 0), it denotes typical “covalent interactions”. For values between 1 and 2 (with ∇^2^*ρ* > 0 and *H*_b_ < 0), the interactions are characterized as having an “intermediate character”. The observation that |*V*_b_|/*G*_b_ > 1 for the CuIn, InCu, Cu, and In clusters clearly indicates that the bonds are not purely closed-shell. Instead, this suggests that there is a significant covalent contribution that must be considered to fully explain the nature of the bonding.

The electron density (*ρ*_b_) at the bond critical points (BCPs) varies significantly across different clusters, reflecting the nature of their bonding interactions. For instance, in the monometallic Cu clusters, the Cu–Cu interactions exhibit relatively high electron density values, with Cu_2_ showing *ρ*_b_ = 0.047 eÅ^−3^, Cu_3_ displaying *ρ*_b_ values between 0.028 and 0.043 eÅ^−3^, and Cu_4_ reaching up to 0.044 eÅ^−3^. These values, combined with the relatively large positive Laplacian values (up to 0.182 eÅ^−5^ for Cu_4_), indicate metallic bonding, where electron delocalization is dominant. In contrast, In–In interactions in indium clusters exhibit lower electron density values, such as *ρ*_b_ = 0.023 eÅ^−3^ for In_3_ and *ρ*_b_ = 0.027 eÅ^−3^ for In_6_, suggesting weaker bonding and a more ionic-like character, consistent with the lower cohesive energy of bulk indium. In bimetallic Cu–In clusters, the Cu–In interactions demonstrate an intermediate character between metallic and covalent bonding. In Cu_3_In_1_, the Cu–In bonds have ρb values between 0.032 and 0.035 eÅ^−3^, whereas in Cu_5_In_1_, these values increase slightly to 0.037–0.038 eÅ^−3^, indicating stronger Cu–In interactions in larger clusters. The Laplacian values (∇^2^*ρ*_b_) for Cu–In bonds are consistently positive, ranging from 0.060 to 0.075 eÅ^−5^, confirming a closed-shell interaction typical of ionic or metallic bonding. However, the total electronic energy density (*H*_b_) at the BCPs is slightly negative (−0.005 to −0.007 he^−1^), signifying a significant covalent contribution to the bonding.

Further classification using the ratio of potential energy density (*V*_b_) to kinetic energy density (*G*_b_), |*V*_b_|/*G*_b_, reveals a more detailed picture of the bonding nature. For Cu_3_In_1_, this ratio ranges from 1.250 to 1.273, while for Cu_5_In_1_, it is slightly higher, around 1.269 to 1.280, confirming a mixed bonding character. In Cu_7_In_1_, the Cu–In bonds have slightly lower ρb values (∼0.031–0.032 eÅ^−3^), coupled with smaller Laplacian values (∼0.064–0.067 eÅ^−5^), suggesting a shift towards more metallic-like interactions in larger bimetallic clusters. The Cu–Cu interactions in these systems retain a primarily metallic character, as seen from the relatively high ρb values (∼0.036–0.041 eÅ^−3^ in Cu_5_In_1_ and Cu_7_In_1_) and larger Laplacians (∼0.120–0.134 eÅ^−5^), indicative of delocalized electron density.

These results indicate that while pure Cu clusters exhibit predominantly metallic bonding and In clusters show ionic-like interactions, the Cu–In clusters display a mixed bonding nature with significant covalent contributions. The presence of covalency in Cu–In interactions enhances charge transfer and cluster stability, which can play a crucial role in tuning the electronic and catalytic properties of Cu–In nanoparticles. This comprehensive QTAIM analysis provides a deeper understanding of how bonding interactions evolve with cluster size and composition, further reinforcing the structural and energetic stability of these systems.

### 3.4. Catalytic relevance

The catalytic performance of Cu–In clusters in CO_2_ reduction is closely linked to their structural and electronic properties, which influence adsorption strength, charge transfer, and reaction pathways. Density Functional Theory (DFT) calculations reveal that Cu-rich clusters exhibit a higher density of states near the Fermi level, which enhances electron donation to CO_2_, facilitating its activation. The presence of In, on the other hand, modifies the electronic structure by reducing the d-band center of Cu, thereby tuning the adsorption strength of reaction intermediates such as CO_2_, CO, and HCOO. In Cu-rich clusters (*e.g.*, Cu_3_In_1_, Cu_5_In_1_), the higher electron density at Cu sites promotes direct CO_2_ activation *via* a bent adsorption mode, favoring the formation of CO as a key intermediate. These clusters tend to favor pathways leading to CO desorption or further hydrogenation to hydrocarbons. Conversely, In-rich clusters (*e.g.*, In_3_Cu_1_, In_5_Cu_1_) exhibit a stronger interaction with oxygenated species, stabilizing HCOO intermediates and making the formate pathway more favorable, which is associated with selective CO_2_ reduction to methanol or formic acid. The potential active sites within these clusters are primarily the Cu–In bridge sites, where electron density redistribution facilitates optimal CO_2_ adsorption. Molecular orbital analysis suggests that In atoms act as electron reservoirs, donating charge to adjacent Cu sites, thereby enhancing catalytic activity. Additionally, the mixed bonding character (as revealed by QTAIM analysis) ensures a balance between stability and reactivity, a crucial factor in catalyst design. Overall, the composition-dependent electronic tuning of Cu–In clusters enables selective control over CO_2_ reduction pathways, offering a promising strategy for optimizing catalyst performance. Future experimental validation of these theoretical findings could further elucidate their practical applications in sustainable CO_2_ conversion technologies.

## 4. Conclusions

The DFT-based Mexican Enhanced Genetic Algorithm (MEGA-DFT) approach has been successfully applied to search for the global minima of gas-phase Cu, In, and Cu–In clusters with sizes of *N* = 2–13 atoms for monometallic clusters and *N* = 2–10 for bimetallic clusters. Local DFT minimizations of metal atoms and dimers have also been computed. The GMs of the free Cu clusters (*N* = 2–6) are found to be 2D structures, and Cu clusters with sizes greater than 6 are found to be 3D structures. Indium clusters are found to be a 2D structure for *N* = 2–4, and sizes greater than 4 are found to be 3D structures. It is worth noting that Cu atoms are suggested to show a high tendency to locate at interior high-coordinate sites, while In atoms prefer low-coordinate sites, the reasons for this is might be the bigger atomic radius of In as well as its low surface energy and its higher electronegativity. Based on our calculations of mixing and binding energies, this conclusion has been confirmed. Generally, the negative mixing trend for Cu–In clusters was found between 0.0 and −3.9 eV, but the maximum mixing tendency is achieved for size *N* = 5 in cluster In_3_Cu_2_ (−3.94 eV). These results confirm that In atoms prefer low-coordinated sites, such as the edges. Moreover, the positive peaks in Δ_2_*E* graph and the high values of *Δ*_HL_, resulted in higher relative stabilities for even-number Cu clusters, in comparison with the odd-number neighboring clusters, this has confirmed the enhanced stability of closed-shell even-electron clusters of Cu due to the contribution of one 4s electron per atom to the delocalized cluster bonding. As for bimetallic CuIn, there is a difference in the stability pattern when a single In atom is incorporated into the pure Cu clusters. Clearly, In-doped Cu clusters display a reversed stability pattern, showing an odd–even trend of stable-unstable. For both pure clusters, the highest stability, indicated by positive peaks in Δ_2_*E*, occurs at *N* = 8, whereas the highest stability is observed at *N* = 7 and *N* = 6 for In-doped Cu clusters and Cu-doped In clusters, respectively. A comparison of the HSE06 spin-projected band structure and the PDOS of bimetallic Cu_7_In_1_ with pure Cu results in the conclusion that the presence of In atom affects the electronic structure of bimetallic Cu–In and causes in a reduction of the band gap. By analyzing the topological parameters of Cu–Cu, In–In and Cu–In bonds of bimetallic clusters using The Quantum Theory of Atoms in Molecules (QTAIMs), the results indicate that the interactions are not purely closed-shell and there is a significant covalent contributions that must be considered.

## Data availability

The data supporting this article have been included as part of the ESI.†

## Author contributions

Norah O. Alotaibi: conceptualization, MEGA-DFT simulations, formal analysis, data curation, investigation, methodology, visualization, and writing – original draft, writing – review & editing. Heider A. Abdulhussein: conceptualization, supervision, validation, formal analysis, data curation, investigation, methodology, writing – review & editing. Shatha M. Alamri: performed electronic structure calculations at HSE06 level, formal analysis, visualization, and writing – original draft. Noorhan Ali Hamza: performed topological analysis, formal analysis, visualization, and writing – original draft. Abbas H. Abo Nasria: data curation, investigation, methodology, writing – review & editing.

## Conflicts of interest

There are no conflicts to declare.

## Supplementary Material

RA-015-D4RA07404A-s001
