# Supplementary material for: Computational insights into the physico-chemical properties of pure and single-atom copper–indium sub-nanometre clusters: a DFT-genetic algorithm approach
Source: RSC Adv. 2025 Feb 20;15(8):5856–75. doi: 10.1039/d4ra07404a (PMC11841672; doi:10.1039/d4ra07404a)
Supplement: RA-015-D4RA07404A-s001 [file RA-015-D4RA07404A-s001.pdf]

Supplementary Information (SI) for RSC Advances.
This journal is © The Royal Society of Chemistry 2025

Supporting Information

Computational insights into the physco-chemical properties of pure and single-atom copper-indium sub-nanometre clusters: a DFT-genetic algorithm approach

Norah O. Alotaibi[1], Heider A. Abdulhussein[2,3], Shatha M. Alamri[1], Noorhan Ali Hamza[4], Abbas H. Abo Nasria[5]*

[1]Chemistry Department, Faculty of Science, King Abdulaziz University, Jeddah, 21589, Saudi Arabia

[2]Department of Chemistry, Faculty of Science, University of Kufa, Najaf, Iraq

[3]College of Engineering, University of Warith Al-Anbiyaa, Kerbala, Iraq

[4]Department of Chemistry, Faculty of Education for Pure Science, University of Kerbala, Kerbala, Iraq

[5]Department of Physics, Faculty of Science, University of Kufa, Najaf, Iraq.

Corresponding Author:

Heider A. Abdulhussein, *Email: haydera.abdulhussein@uokufa.edu.iq

Content

1. **Average distances.**
2. **Energies, point groups, and the optimal spin states and average distances.**
3. **Energies of Cu and In and GM of bimetallic systems:**
4. **Band gaps, PDOS, TDOS, and band structures of clusters calculated at the HSE06 level.**
5. **Calculated quantum molecular descriptors for CuIn systems**
6. **Topological parameters**
7. **Molecular graphs for Cu, In, and binary CuIn systems**
8. **Gg**
9. **The convex hull diagram for the Cu-In clusters**
10. **XYZ coordinates of minimum-energy structures.**

1. Average distances.

Table S1: Average distances for Cu–Cu and In–In bonds for the Cu_2 and In_2 dimers compared with experimental and theoretical data obtained from literature.

Cu_2	Distance (Å)	In_2	Distance (Å)
**Exp.**	2.25[1]	**Exp.**	3.07[2]
**Theo.**	2.22 [3]	**Theo.**	2.91[4]
**Ours**	2.22	**Ours**	3.06

2. Energies, point groups, and the optimal spin states and average distances.

Table S2: The energies, point groups, and optimal spin states of mono- and bimetallic clusters.

Cluster	Energy (eV)	Point Group	2S+1
Cu_2	-2.753	C_{2v}	singlet
Cu_3	-4.431	C_{2v}	doublet
Cu_4	-7.350	D_{2h}	singlet
Cu_5	-9.856	C_{2v}	doublet
Cu_6	-12.969	D_{3h}	singlet
Cu_7	-16.006	D_{2d}	doublet
Cu_8	-19.095	C_s	singlet
Cu_9	-21.454	D_{2d}	doublet
Cu_{10}	-24.589	C_s	singlet
Cu_{11}	-27.341	C_1	doublet
Cu_{12}	-30.491	C_{2v}	singlet
Cu_{13}	-33.373	C_{2v}	doublet
In_2	-1.815	C_{2v}	triplet
In_3	-3.711	C_{2v}	quartet
In_4	-5.951	D_{4h}	triplet
In_5	-7.966	C_{2v}	doublet
In_6	-10.607	C_s	doublet
In_7	-13.158	C_s	doublet
In_8	-15.596	C_s	singlet
In_9	-17.364	C_s	doublet
In_{10}	-19.838	C_s	singlet
In_{11}	-22.212	C_s	doublet
In_{12}	-24.674	C_s	singlet
In_{13}	-27.228	C_s	doublet
CuIn	-2.9122	$C_{\infty v}$	singlet

Cu_2In	-5.0152	C_{2v}	doublet
Cu_3In	-7.8788	C_{2v}	singlet
Cu_4In	-10.423	C_{3v}	doublet
Cu_5In	-13.450	C_{3v}	doublet
Cu_6In	-15.749	C_s	doublet
Cu_7In	-18.599	C_s	doublet
Cu_8In	-20.616	C_s	doublet
Cu_9In	-24.407	C_s	singlet
In_2Cu	-4.681	C_{2v}	doublet
In_3Cu	-6.748	C_{2v}	triplet
In_4Cu	-9.046	C_{3v}	doublet
In_5Cu	-11.012	C_{3v}	singlet
In_6Cu	-13.853	D_{4h}	singlet
In_7Cu	-16.182	C_s	singlet
In_8Cu	-18.421	C_s	singlet
In_9Cu	-20.628	C_s	singlet

Table S3: Average bond distances of Cu-Cu, In-In, and Cu-In (in Å).

**Cluster**	**Average Bond Distance (Å)**		
	**Cu-Cu**	**In-In**	**Cu-In**
Cu_2	2.225	-	-
Cu_3	2.277	-	-
Cu_4	2.366	-	-
Cu_5	2.353	-	-
Cu_6	2.345	-	-
Cu_7	2.420	-	-
Cu_8	2.394	-	-
Cu_9	2.458	-	-
Cu_{10}	2.443	-	-
Cu_{11}	2.471	-	-
Cu_{12}	2.369	-	-
Cu_{13}	2.404	-	-
In_2	-	3.062	-
In_3	-	3.661	-
In_4	-	2.975	-
In_5	-	2.950	-
In_6	-	2.928	-
In_7	-	3.048	-
In_8	-	3.093	-
In_9	-	2.935	-
In_{10}	-	3.035	-
In_{11}	-	3.012	-
In_{12}	-	3.271	-
In_{13}	-	2.974	-
CuIn	-	-	2.534
Cu_2In	2.309	-	2.645
Cu_3In	2.306	-	2.640
Cu_4In	2.374	-	2.715
Cu_5In	2.385	-	2.602

Cu_6In	2.406	-	2.692
Cu_7In	2.406	-	2.663
Cu_8In	2.442	-	2.667
Cu_9In	2.413	-	2.634
In_2Cu	-	3.210	2.649
In_3Cu	-	3.057	2.598
In_4Cu	-	3.079	2.754
In_5Cu	-	2.990	2.696
In_6Cu	-	2.924	2.716
In_7Cu	-	3.012	2.702
In_8Cu	-	3.150	2.711
In_9Cu	-	3.168	2.628

3. Energies of Cu and In and GM of bimetallic systems:

Table S4: Energies, binding energies, excess energies and second difference in energy for all compositions of CuIn systems, N=2-8.

**Composition**	**E_b /e V**	**Δ/e V**	**Δ_2E /e V**
**N=2**			
Cu_2	1.37	0.00	---
Cu_1In_1	1.45	-0.94	---
In_2	1.31	0.00	---
**N=3**			
Cu_3	2.24	0.00	-1.24
In_1Cu_2	1.97	-0.82	-0.76
In_2Cu_1	1.40	-0.73	-0.29
In_3	1.84	0.00	-0.34
**N=4**			
Cu_4	2.85	0.00	0.41
In_1Cu_3	1.82	-0.87	0.31
In_2Cu_2	1.93	-1.18	---
In_3Cu_1	1.77	-0.44	-0.23
In_4	2.30	0.00	0.22
**N=5**			
Cu_5	3.24	0.00	-0.60
In_1Cu_4	1.92	-0.94	-0.48
In_2Cu_3	1.97	-3.43	---
In_3Cu_2	2.55	-3.94	---
In_4Cu_1	1.92	-0.70	0.33
In_5	2.61	0.00	-0.62
**N=6**			
Cu_6	3.68	0.00	0.07
In_1Cu_5	2.06	-0.87	0.72
In_2Cu_4	2.13	-1.22	---
In_3Cu_3	2.08	-0.88	---
In_4Cu_2	2.03	-0.49	---
In_5Cu_1	1.96	-0.01	-0.87
In_6	2.99	0.00	0.08

**N=7**			
Cu_7	4.06	0.00	-0.05
In_1Cu_6	2.06	-0.14	0.83
In_2Cu_5	2.11	-0.48	---
In_3Cu_4	2.15	-0.68	---
In_4Cu_3	2.11	-0.35	---
In_5Cu_2	2.07	-1.95	---
In_6Cu_1	2.11	-0.28	0.51
In_7	3.30	0.00	0.11
**N=8**			
Cu_8	4.42	0.00	0.73
In_1Cu_7	2.12	0.05	0.83
In_2Cu_6	2.19	-0.48	---
In_3Cu_5	2.20	-0.49	---
In_4Cu_4	2.20	-0.48	---
In_5Cu_3	2.18	0.05	---
In_6Cu_2	2.24	-0.75	---
In_7Cu_1	2.16	-0.14	-0.11
In_8	3.37	0.00	0.66

4. Band gaps, PDOS, TDOS calculated at the HSE06 level.

Table S5: Band gaps of mono- and bimetallic clusters.

**Clusters**	**Cu_2**	**Cu_3**	**Cu_4**	**Cu_5**	**Cu_6**	**Cu_7**	**Cu_8**
**Band gap (eV)**	3.12	0.881	1.724	1.124	3.078	0.998	2.393
**Clusters**	**In_2**	**In_3**	**In_4**	**In_5**	**In_6**	**In_7**	**In_8**
**Band gap (eV)**	0.734	1.168	0.791	0.881	0.995	0.963	1.274
**Clusters**	**In_4Cu**		**Cu_3In**		**Cu_5In**	**Cu_7In**	
**Band gap (eV)**	0.674		2.868		2.521	2.103	

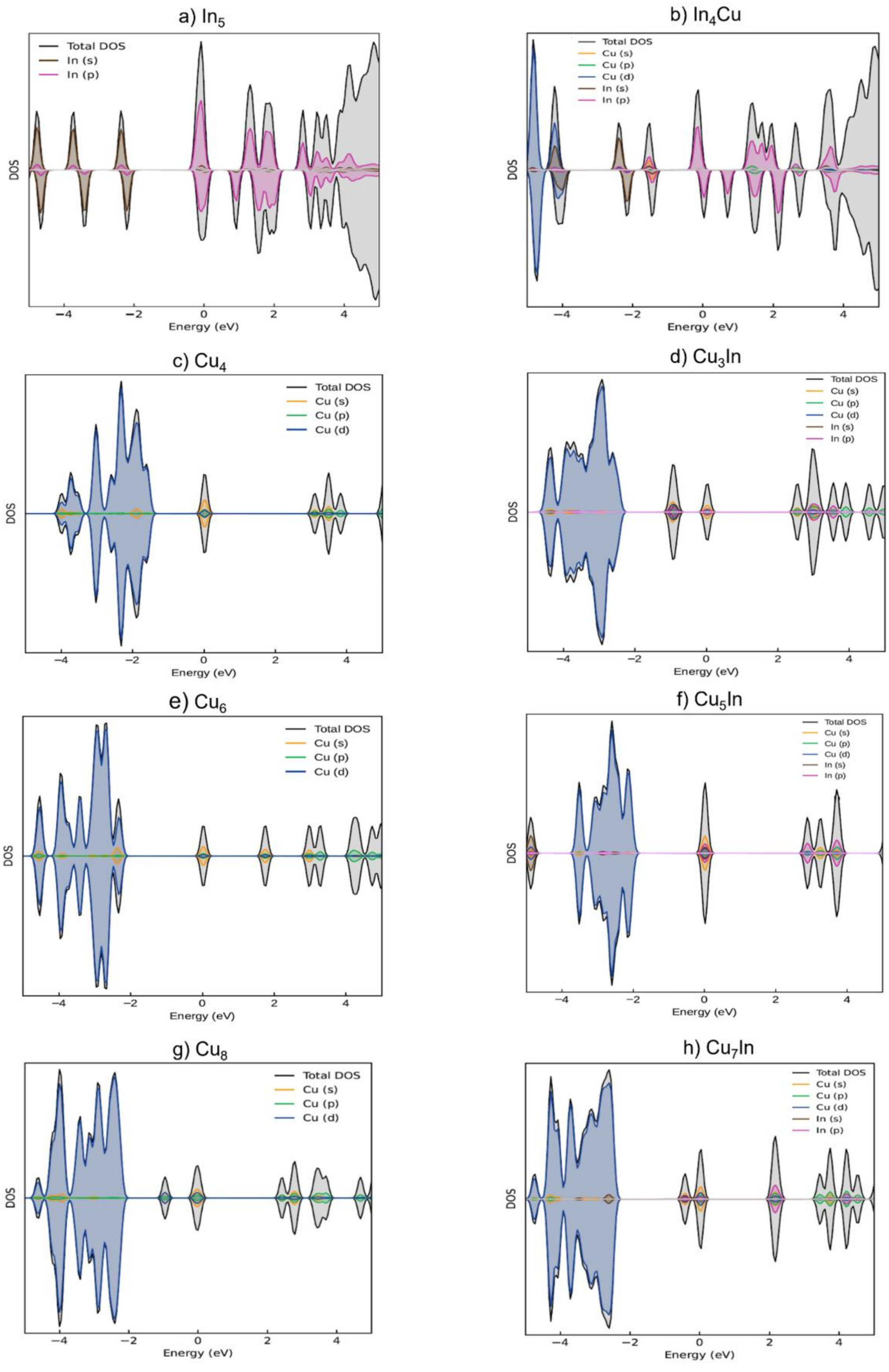

Figure S1: Partial and total density of state.

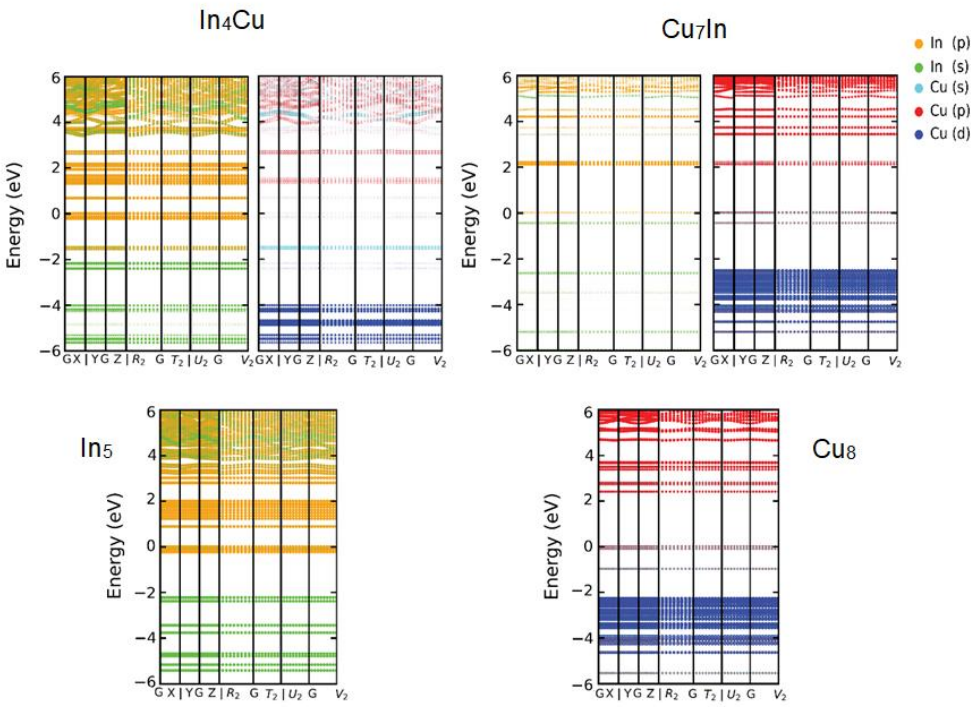

Figure S2: Projected band structures of bimetallic a) In_4Cu b) Cu_7In and monometallic c) In_5 and d) Cu_8, at HSE06 level.

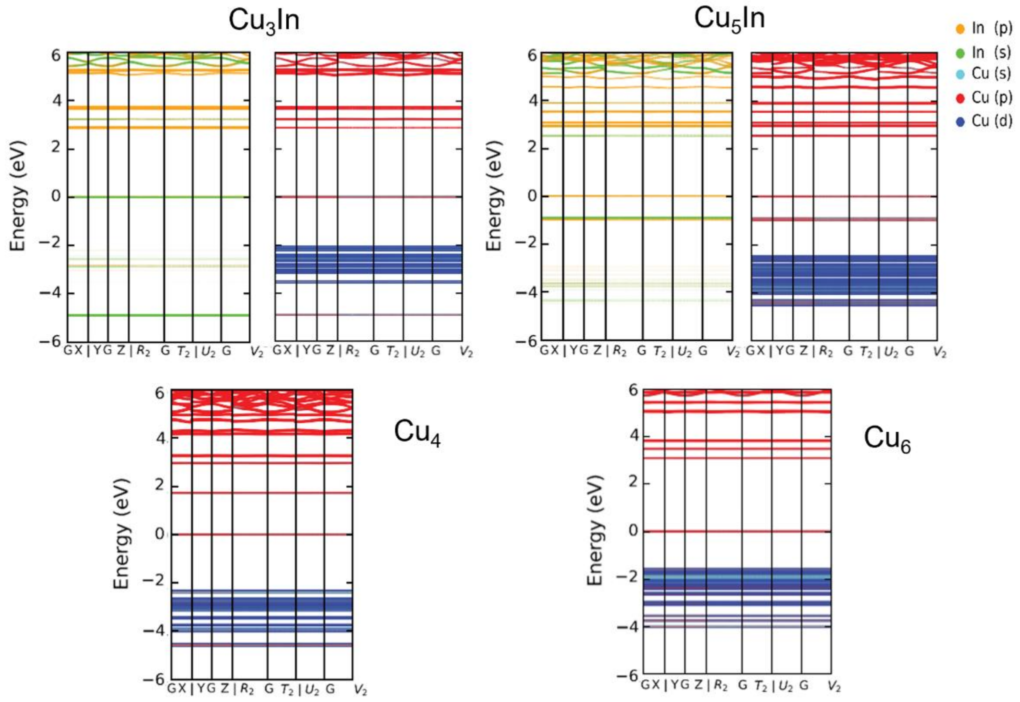

Figure S3: Projected band structures of bimetallic a) In_3Cu b) Cu_3In and monometallic c) Cu_4 and d) Cu_6, at HSE06 level.

5. Calculated quantum molecular descriptors for CuIn systems

Table S6: Calculated quantum molecular descriptors for atoms, dimers and clusters (N = 2-5).

Cluster	Δ_{HL}	I/eV	A/e V	χ/eV	μ/eV	η/eV	S /eV	ω/eV
**N=2**								
In_2	1.92	2.53	6.65	4.79	-4.79	-0.12	-8.33	-95.60
Cu_1In_1	1.70	3.47	4.18	3.33	-3.33	-0.70	-1.42	-7.92
Cu_2	1.56	3.02	4.56	3.78	-3.78	-1.56	-0.64	-4.57
**N=3**								
In_3	0.90	2.75	3.66	1.93	-1.93	-0.90	-1.11	-2.06
Cu_2In_1	1.50	4.44	4.94	4.19	-4.19	-0.50	-2.00	-17.55
Cu_3	1.48	3.22	4.70	3.96	-3.96	-1.48	-0.67	-5.29
**N=4**								
In_4	0.26	3.21	3.47	1.82	-1.82	-0.26	-3.84	-6.37
Cu_3In_1	1.30	3.73	4.03	3.38	-3.38	-0.30	-3.33	-19.04
Cu_4	0.94	3.42	4.37	3.89	-3.89	-0.94	-1.06	-8.04
**N=5**								
In_5	0.42	3.18	3.61	1.89	-1.89	-0.42	-2.38	-4.25
Cu_4In_1	1.69	3.45	4.15	3.30	-3.30	-0.69	-1.44	-7.89
Cu_5	0.88	3.77	4.65	4.21	-4.21	-0.88	-1.13	-10.07

6. Topological parameters

Table S7: Topological analysis of selected CuIn, Cu, In Clusters, calculated using the B3LYP/WTBS Method.

Bond	ρ_b(eÅ^{-3})	$\nabla^2\rho_b$(eÅ^{-5})	G_b(he^{-1})	H_b(he^{-1})	V(he^{-1})	V/G
			**Cu_3In**			
Cu1-In	0.034	0.067	0.022	-0.006	-0.028	1.273
Cu2-In	0.032	0.06	0.02	-0.005	-0.025	1.250
Cu3-In	0.035	0.07	0.023	-0.006	-0.029	1.261
Cu1-Cu2	0.041	0.154	0.047	-0.008	-0.055	1.170
Cu2-Cu3	0.04	0.147	0.044	-0.008	-0.052	1.182
			**Cu_5In**			
Cu1-In	0.038	0.075	0.026	-0.007	-0.033	1.269
Cu3-In	0.038	0.073	0.025	-0.007	-0.032	1.280
Cu4-In	0.037	0.073	0.025	-0.007	-0.032	1.280
Cu5-In	0.038	0.074	0.025	-0.007	-0.032	1.280

Cu1-Cu2	0.036	0.13	0.039	-0.006	-0.045	1.154
Cu1-Cu3	0.03	0.099	0.029	-0.005	-0.034	1.172
Cu1-Cu4	0.03	0.097	0.029	-0.004	-0.033	1.138
Cu2-Cu3	0.036	0.13	0.039	-0.007	-0.046	1.179
Cu2-Cu4	0.035	0.123	0.037	-0.006	-0.043	1.162
Cu2-Cu5	0.037	0.134	0.04	-0.007	-0.047	1.175
Cu3-Cu5	0.029	0.091	0.027	-0.004	-0.031	1.148
Cu4-Cu5	0.03	0.099	0.029	-0.005	-0.034	1.172
**Cu_7In**						
Cu1-In	0.031	0.065	0.021	-0.005	-0.026	1.238
Cu2-In	0.032	0.067	0.022	-0.005	-0.027	1.227
Cu3-In	0.031	0.064	0.021	-0.005	-0.025	1.190
Cu1-Cu2	0.03	0.097	0.029	-0.005	-0.033	1.138
Cu1-Cu3	0.03	0.097	0.029	-0.004	-0.033	1.138
Cu1-Cu4	0.036	0.118	0.036	-0.007	-0.043	1.194
Cu1-Cu6	0.036	0.119	0.037	-0.007	-0.043	1.162
Cu2-Cu3	0.029	0.096	0.028	-0.004	-0.033	1.179
Cu2-Cu4	0.036	0.119	0.036	-0.007	-0.043	1.194
Cu2-Cu5	0.036	0.119	0.036	-0.007	-0.043	1.194
Cu3-Cu5	0.036	0.12	0.037	-0.007	-0.043	1.162
Cu3-Cu6	0.036	0.118	0.036	-0.007	-0.043	1.194
Cu4-Cu5	0.032	0.106	0.032	-0.005	-0.037	1.156
Cu4-Cu6	0.031	0.105	0.031	-0.005	-0.036	1.161
Cu4-Cu7	0.035	0.119	0.036	-0.006	-0.042	1.167
Cu5-Cu6	0.031	0.102	0.03	-0.005	-0.035	1.167
Cu5-Cu7	0.035	0.117	0.035	-0.006	-0.041	1.171
Cu6-Cu7	0.036	0.121	0.037	-0.006	-0.043	1.162
**Cu_2**						
Cu1-Cu2	0.047	0.159	0.049	-0.01	-0.059	1.204
**Cu_3**						
Cu1-Cu2	0.043	0.155	0.047	-0.008	-0.056	1.180
Cu1-Cu3	0.043	0.154	0.047	-0.008	-0.056	1.180
Cu2-Cu3	0.028	0.085	0.025	-0.004	-0.029	1.151
**Cu_4**						
Cu1-Cu2	0.044	0.182	0.054	-0.009	-0.063	1.167
Cu1-Cu3	0.037	0.123	0.038	-0.007	-0.044	1.158
Cu1-Cu4	0.037	0.12	0.037	-0.007	-0.043	1.162
Cu2-Cu3	0.036	0.119	0.036	-0.006	-0.043	1.194
Cu2-Cu4	0.037	0.122	0.037	-0.007	-0.044	1.189
**Cu_5**						
Cu1-Cu2	0.036	0.127	0.038	-0.006	-0.045	1.169
Cu2-Cu3	0.037	0.125	0.038	-0.007	-0.045	1.179
Cu2-Cu5	0.037	0.125	0.038	-0.007	-0.045	1.176

Cu1-Cu5	0.040	0.126	0.039	-0.008	-0.047	1.195
Cu1-Cu3	0.036	0.126	0.038	-0.006	-0.044	1.169
Cu1-Cu4	0.041	0.130	0.040	-0.008	-0.048	1.194
Cu3-Cu4	0.037	0.125	0.038	-0.007	-0.045	1.176
**Cu_6**						
Cu1-Cu2	0.036	0.122	0.037	-0.006	-0.043	1.162
Cu1-Cu3	0.035	0.117	0.035	-0.006	-0.042	1.200
Cu1-Cu4	0.04	0.126	0.039	-0.007	-0.047	1.205
Cu1-Cu5	0.04	0.128	0.039	-0.008	-0.047	1.205
Cu2-Cu3	0.036	0.122	0.037	-0.006	-0.043	1.162
Cu2-Cu4	0.039	0.123	0.038	-0.007	-0.045	1.184
Cu2-Cu6	0.039	0.125	0.039	-0.007	-0.046	1.179
Cu3-Cu5	0.04	0.127	0.039	-0.007	-0.047	1.205
Cu3-Cu6	0.04	0.126	0.039	-0.007	-0.047	1.205
**Cu_7**						
Cu1-Cu3	0.027	0.090	0.026	-0.004	-0.030	1.143
Cu1-Cu4	0.035	0.120	0.036	-0.006	-0.042	1.172
Cu3-Cu4	0.034	0.113	0.034	-0.006	-0.040	1.171
Cu4-Cu6	0.033	0.114	0.034	-0.006	-0.040	1.162
Cu1-Cu7	0.034	0.115	0.035	-0.006	-0.041	1.171
Cu2-Cu3	0.034	0.112	0.034	-0.006	-0.040	1.172
Cu5-Cu6	0.034	0.116	0.034	-0.006	-0.040	1.162
Cu3-Cu6	0.034	0.115	0.035	-0.006	-0.041	1.172
Cu2-Cu5	0.033	0.114	0.034	-0.006	-0.040	1.162
Cu3-Cu7	0.035	0.119	0.036	-0.006	-0.042	1.172
Cu2-Cu7	0.033	0.112	0.033	-0.005	-0.039	1.162
Cu3-Cu5	0.034	0.113	0.034	-0.006	-0.040	1.171
Cu1-Cu6	0.034	0.111	0.034	-0.006	-0.039	1.171
**Cu_8**						
Cu1-Cu3	0.027	0.090	0.026	-0.004	-0.030	1.143
Cu1-Cu4	0.035	0.120	0.036	-0.006	-0.042	1.172
Cu3-Cu4	0.034	0.113	0.034	-0.006	-0.040	1.171
Cu4-Cu6	0.033	0.114	0.034	-0.006	-0.040	1.162
Cu1-Cu7	0.034	0.115	0.035	-0.006	-0.041	1.171
Cu2-Cu3	0.034	0.112	0.034	-0.006	-0.040	1.172
Cu5-Cu6	0.034	0.116	0.034	-0.006	-0.040	1.162
Cu3-Cu6	0.034	0.115	0.035	-0.006	-0.041	1.172
Cu2-Cu5	0.033	0.114	0.034	-0.006	-0.040	1.162
Cu3-Cu7	0.035	0.119	0.036	-0.006	-0.042	1.172
Cu2-Cu7	0.033	0.112	0.033	-0.005	-0.039	1.162
Cu3-Cu5	0.034	0.113	0.034	-0.006	-0.040	1.171
Cu1-Cu6	0.034	0.111	0.034	-0.006	-0.039	1.171
**Cu_9**						

Cu1-Cu3	0.034	0.115	0.035	-0.006	-0.04	1.143
Cu1-Cu4	0.033	0.113	0.034	-0.006	-0.04	1.176
Cu1-Cu5	0.038	0.13	0.039	-0.007	-0.046	1.179
Cu1-Cu7	0.035	0.12	0.036	-0.006	-0.042	1.167
Cu1-Cu8	0.035	0.121	0.036	-0.006	-0.043	1.194
Cu2-Cu3	0.034	0.115	0.035	-0.006	-0.04	1.143
Cu2-Cu4	0.034	0.114	0.034	-0.006	-0.04	1.176
Cu2-Cu6	0.038	0.131	0.04	-0.007	-0.047	1.175
Cu2-Cu7	0.035	0.118	0.036	-0.006	-0.042	1.167
Cu2-Cu8	0.035	0.118	0.036	-0.006	-0.042	1.167
Cu3-Cu6	0.033	0.111	0.033	-0.006	-0.039	1.182
Cu3-Cu7	0.036	0.121	0.037	-0.006	-0.043	1.162
Cu4-Cu5	0.034	0.112	0.034	-0.006	-0.039	1.147
Cu4-Cu6	0.034	0.113	0.034	-0.006	-0.04	1.176
Cu4-Cu8	0.036	0.12	0.036	-0.006	-0.043	1.194
Cu5-Cu6	0.03	0.097	0.029	-0.004	-0.033	1.138
Cu7-Cu8	0.027	0.083	0.025	-0.004	-0.028	1.120
**In_3**						
In1-In2	0.023	0.038	0.012	-0.002	-0.014	1.192
In1-In3	0.023	0.038	0.012	-0.002	-0.014	1.188
**In_4**						
In1-In3	0.026	0.026	0.01	-0.004	-0.013	1.300
In1-In4	0.026	0.027	0.01	-0.004	-0.014	1.400
In2-In3	0.026	0.027	0.01	-0.003	-0.014	1.400
In2-In4	0.026	0.026	0.01	-0.004	-0.014	1.400
**In_6**						
In2-In4	0.027	0.033	0.012	-0.004	-0.016	1.310
In3-In6	0.027	0.034	0.012	-0.004	-0.016	1.299
In1-In5	0.027	0.037	0.013	-0.003	-0.016	1.266
In1-In6	0.024	0.024	0.009	-0.003	-0.012	1.350
In4-In6	0.022	0.026	0.009	-0.002	-0.011	1.271
**In_7**						
In2-In3	0.024	0.026	0.009	-0.003	-0.012	1.302
In4-In5	0.025	0.031	0.011	-0.003	-0.014	1.293
In2-In7	0.024	0.027	0.010	-0.003	-0.012	1.300
In4-In4	0.023	0.024	0.009	-0.003	-0.012	1.327
In2-In6	0.018	0.014	0.005	-0.002	-0.007	1.344
In1-In6	0.026	0.033	0.011	-0.003	-0.015	1.285
In3-In5	0.028	0.035	0.013	-0.004	-0.016	1.304
In1-In2	0.018	0.014	0.005	-0.002	-0.007	1.345
**In_8**						
In1-In2	0.021	0.021	0.008	-0.002	-0.01	1.250

In1-In3	0.03	0.045	0.015	-0.004	-0.019	1.267
In1-In5	0.023	0.022	0.008	-0.003	-0.011	1.375
In1-In6	0.023	0.022	0.008	-0.003	-0.011	1.375
In2-In4	0.03	0.045	0.015	-0.004	-0.019	1.267
In2-In5	0.023	0.023	0.009	-0.003	-0.011	1.222
In2-In6	0.023	0.022	0.008	-0.003	-0.011	1.375
In3-In4	0.021	0.02	0.008	-0.002	-0.01	1.250
In3-In7	0.023	0.022	0.008	-0.003	-0.011	1.375
In3-In8	0.023	0.022	0.008	-0.003	-0.011	1.375
In4-In7	0.023	0.023	0.009	-0.003	-0.012	1.333
In4-In8	0.023	0.022	0.008	-0.003	-0.011	1.375
In5-In7	0.028	0.041	0.014	-0.003	-0.017	1.214
In6-In8	0.028	0.041	0.014	-0.003	-0.017	1.214

7. Molecular graphs for Cu, In, and binary CuIn systems

**Cu_3In**	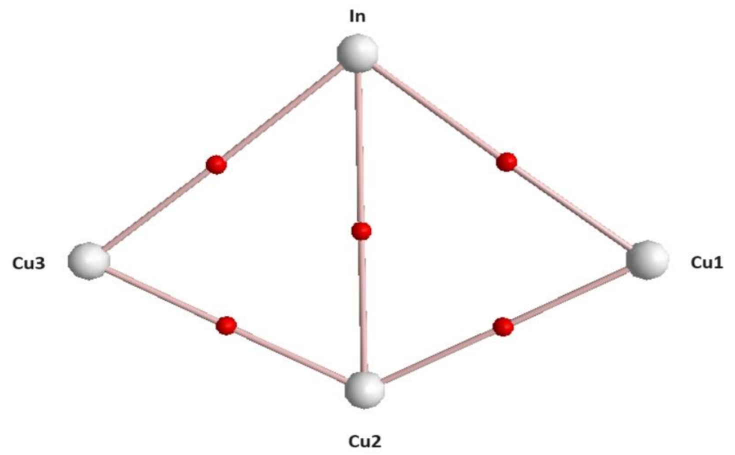
**Cu_5In**	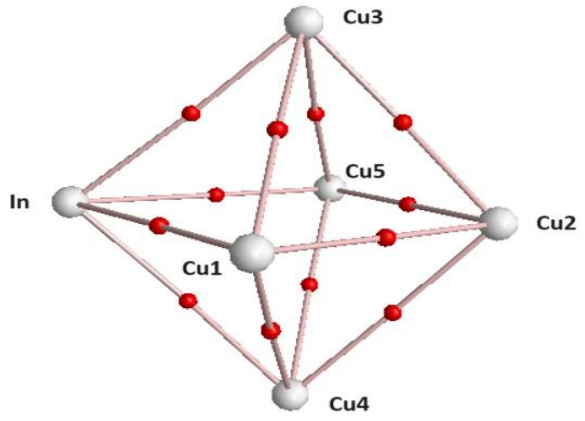

**Cu_7In**	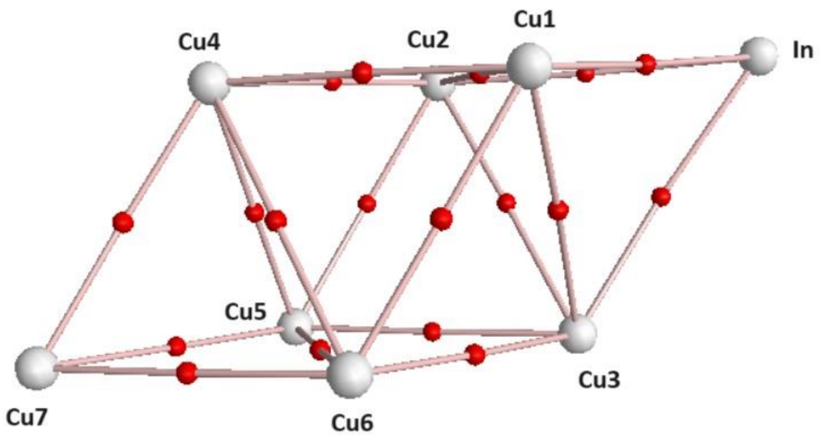
**In_4Cu**	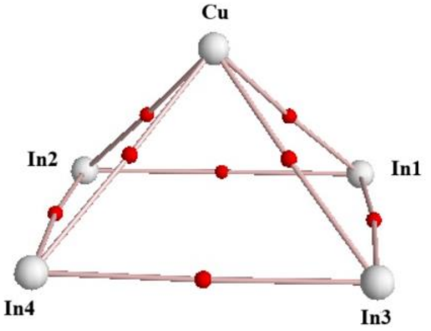
**Cu_2**	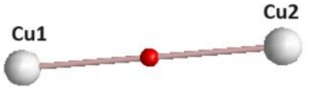
**Cu_3**	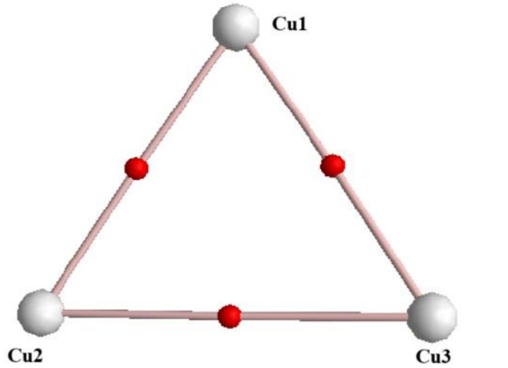

**Cu_4**	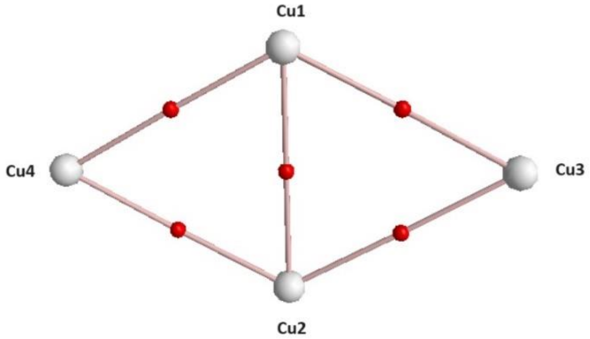
**Cu_5**	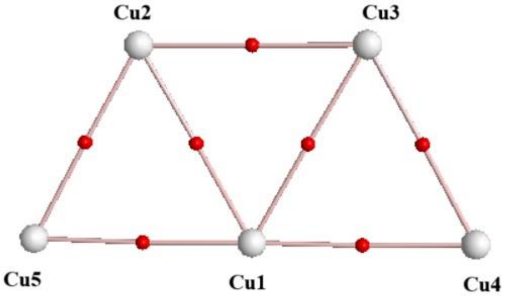
**Cu_6**	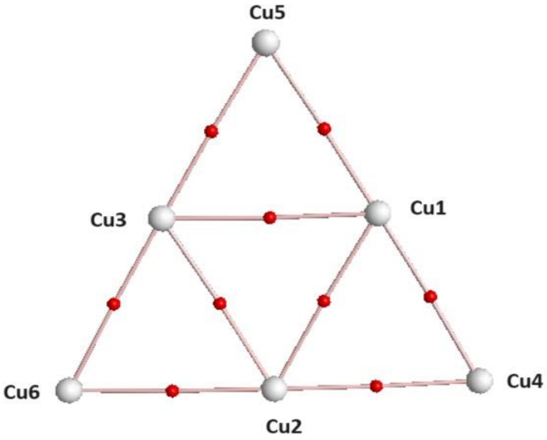
**Cu_7**	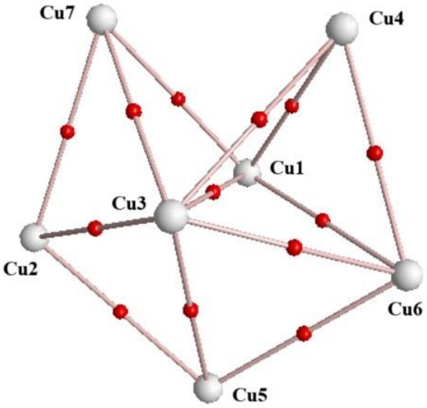

**Cu_8**	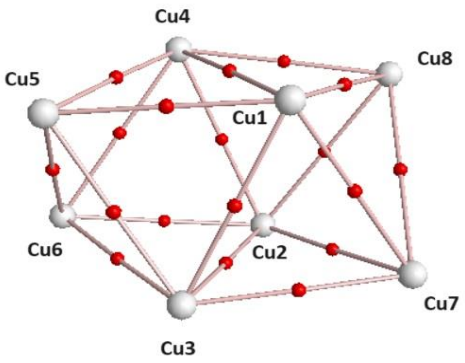
**In_3**	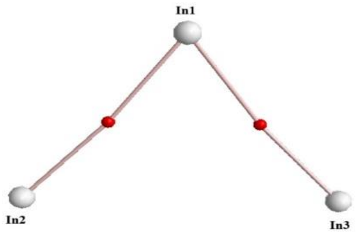
**In_4**	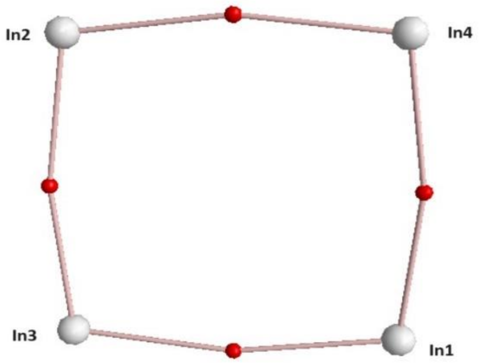
**In_5**	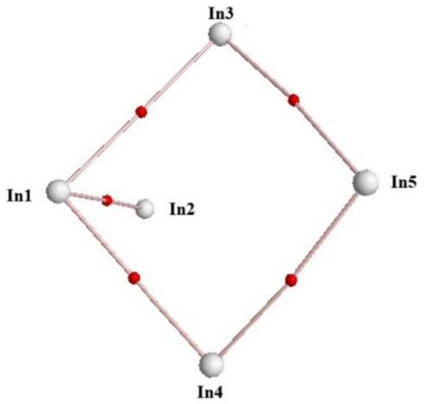

**In_6**	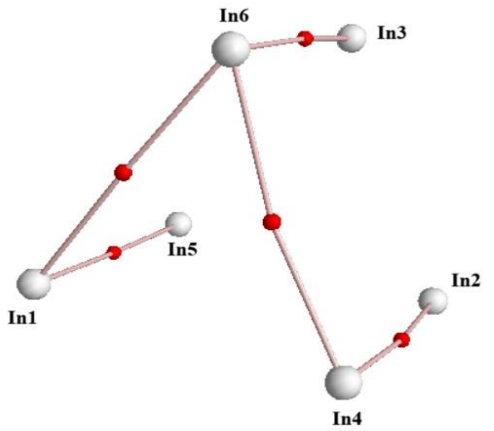
**In_7**	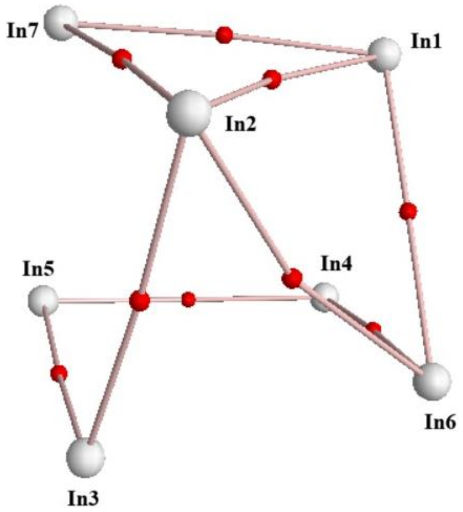
**In_8**	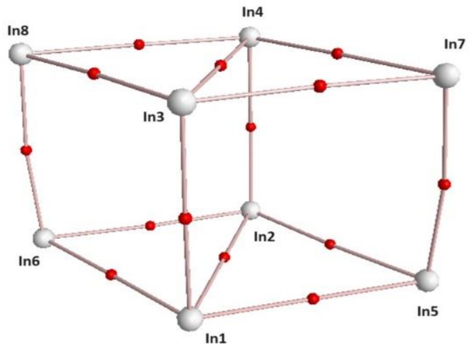

Figure S4: Molecular graph of CuIn, Cu and In Clusters. Solid lines indicate the presence of bond paths, whereas small red dots illustrate the bond critical points.

**Cu_3In**	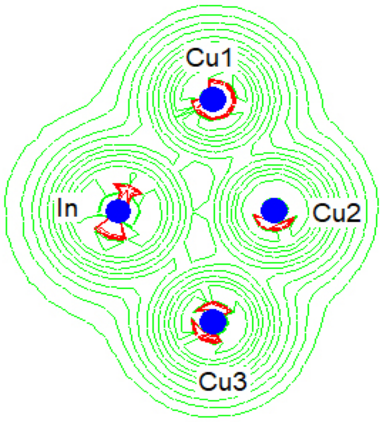
**Cu_5In**	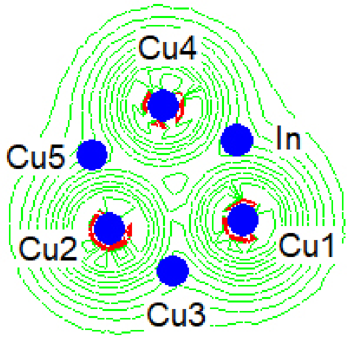
**Cu_7In**	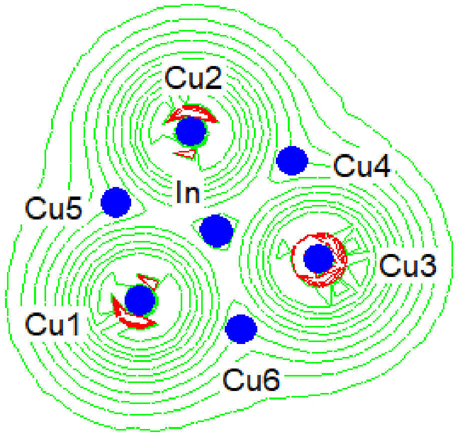

**In_4Cu**	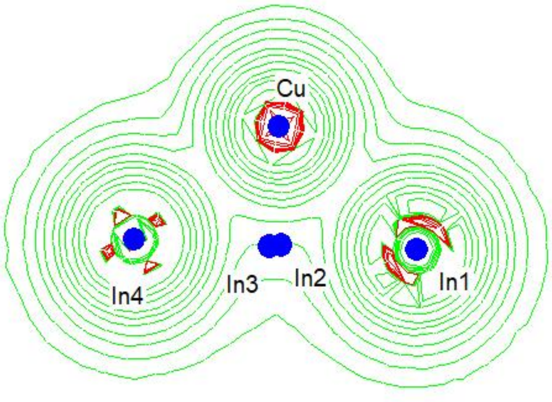
**Cu_2**	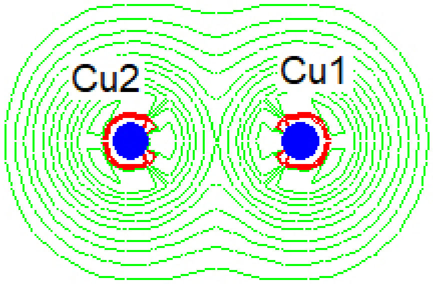
**Cu_3**	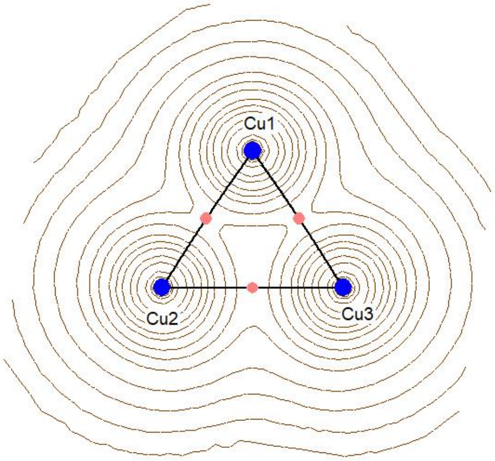

**Cu_4**	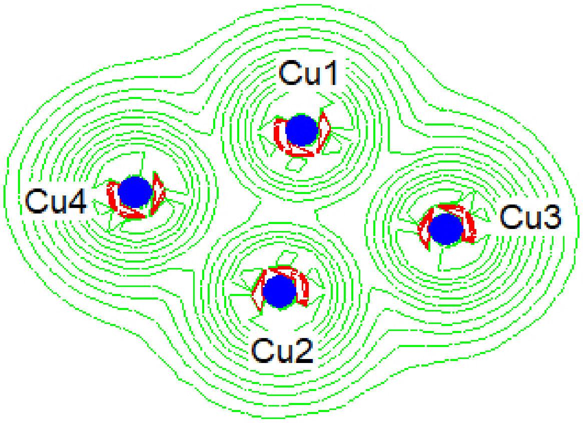Cu1<br>Cu4<br>Cu3<br>Cu2
**Cu_5**	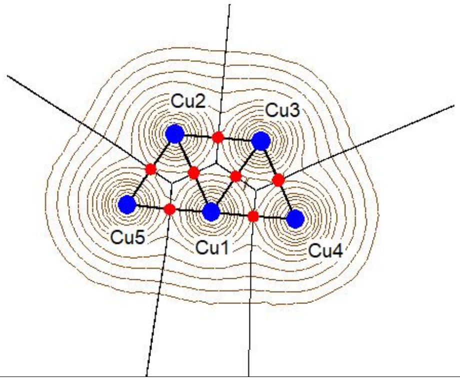Cu2<br>Cu3<br>Cu5<br>Cu1<br>Cu4
**Cu_6**	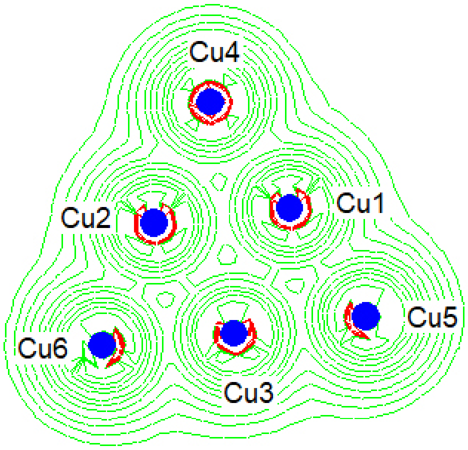Cu4<br>Cu2<br>Cu1<br>Cu6<br>Cu3<br>Cu5

**Cu_7**	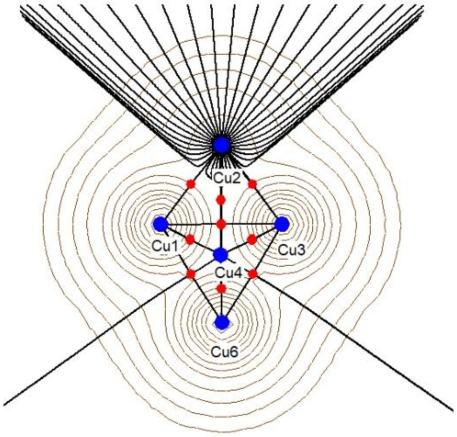 Cu2<br>Cu1<br>Cu3<br>Cu4<br>Cu6
**Cu_8**	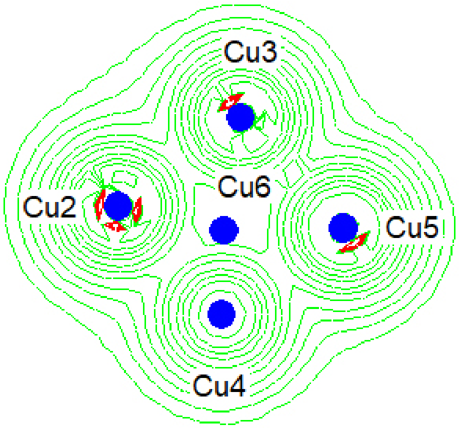 Cu3<br>Cu6<br>Cu2<br>Cu5<br>Cu4
**In_3**	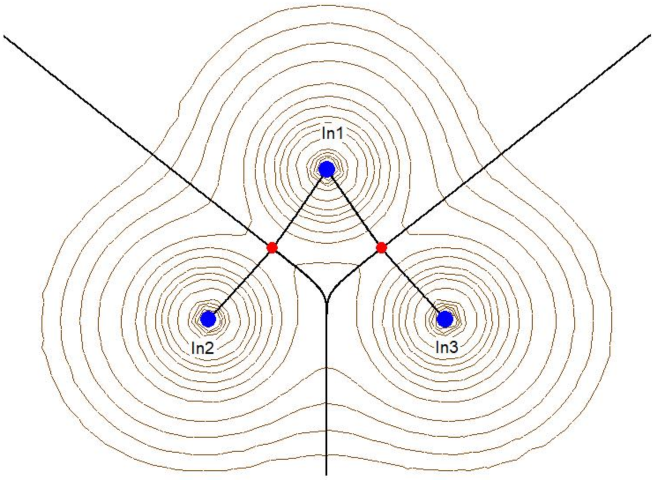 In1<br>In2<br>In3

In_4	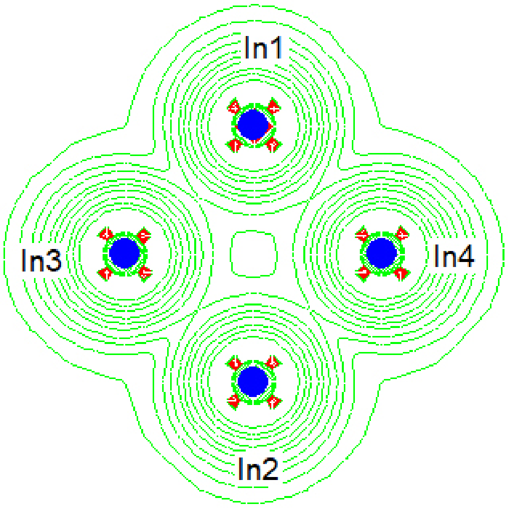In1<br>In3<br>In4<br>In2
In_5	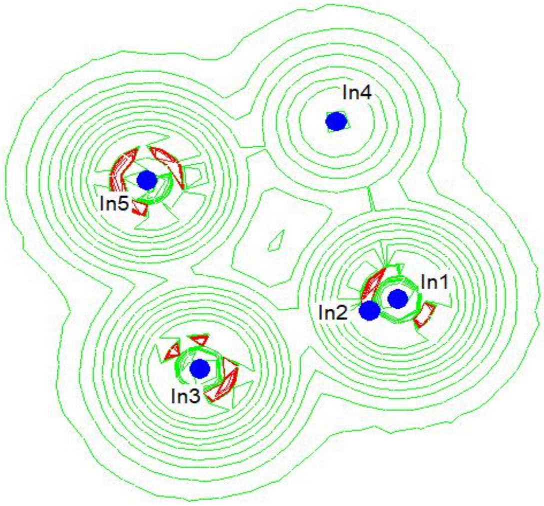In4<br>In5<br>In1<br>In2<br>In3
In_6	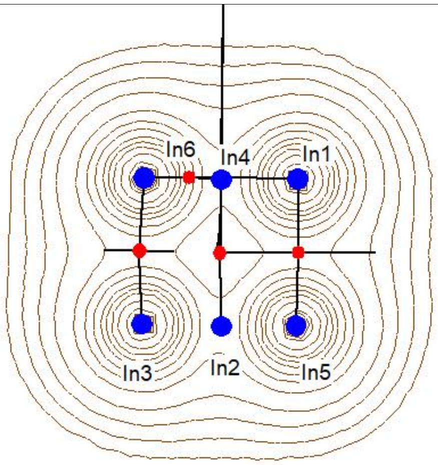In6<br>In4<br>In1<br>In3<br>In2<br>In5

**In$_7$**	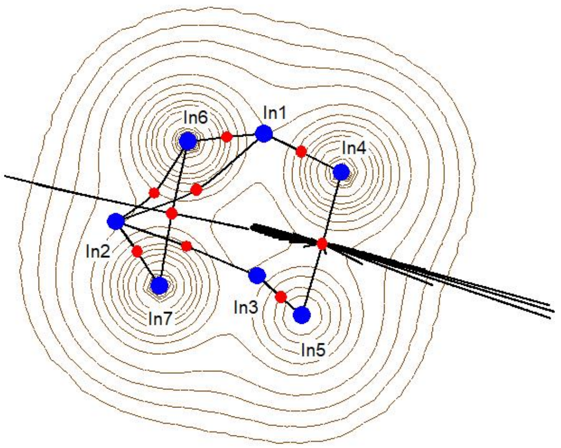
**In$_8$**	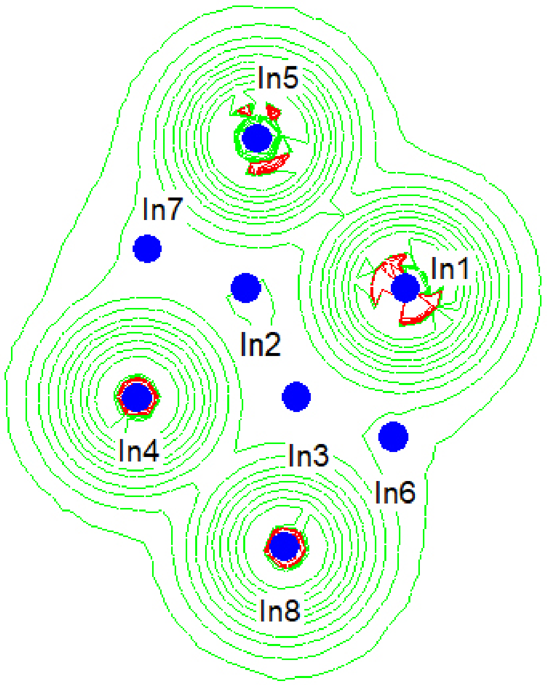

Figure S5: Laplacian maps of the electron density for clusters.

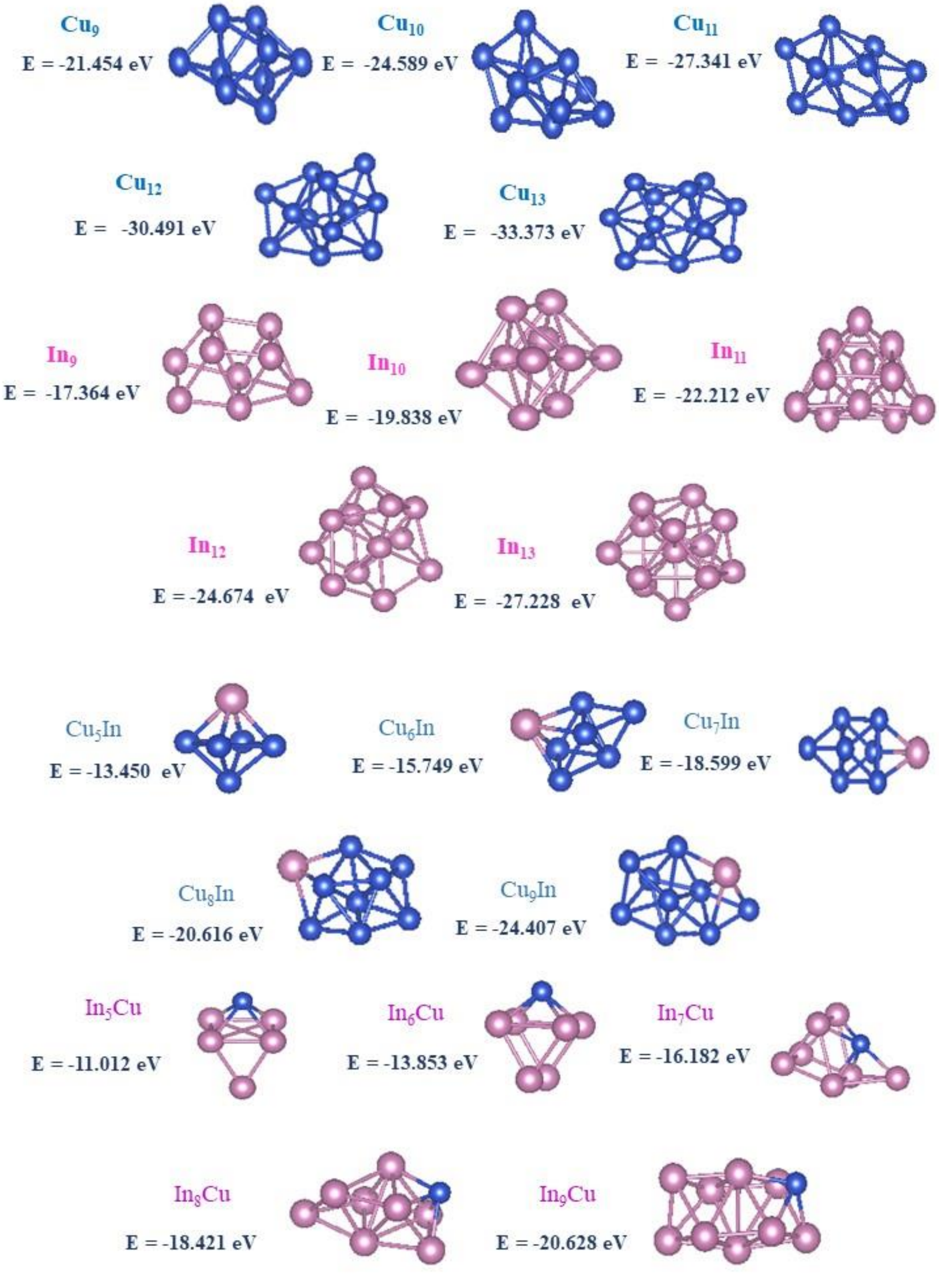

Figure S6: 5 lowest energy configurations of each system and the corresponding energies (in eV).

8. the convex hull diagram for the Cu-In clusters

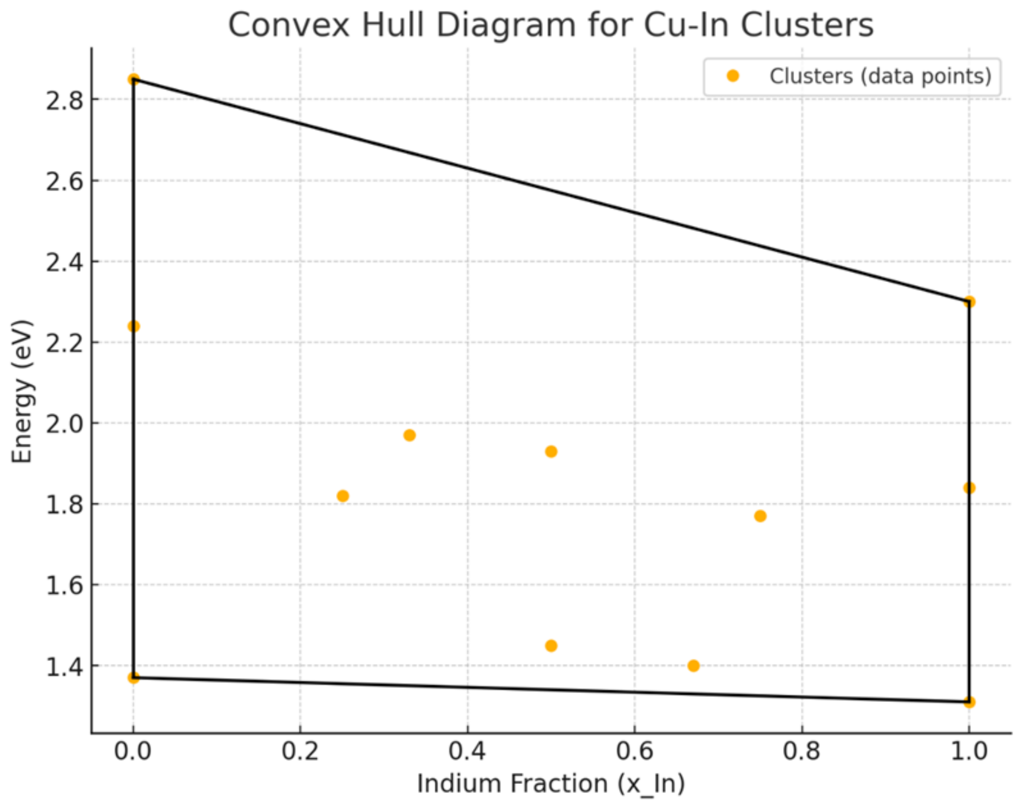

Figure S7: the convex hull diagram for the Cu-In clusters. The x-axis represents the indium fraction (X_{In}), and the y-axis represents the energy (eV). The points connected by solid lines form the convex hull, indicating the thermodynamically stable configurations. Clusters lying on the convex hull are stable, while those above are less stable or metastable.

9. XYZ coordinates of minimum-energy structures

9.1 monometallic Cu

Cu_2

Cu 5.195465 5.298632 5.483022

Cu 6.718685 6.615519 6.431128

Cu_3

Cu 5.999661 5.619271 7.054590

Cu 7.681839 7.105236 7.439252

Cu 6.210180 7.167040 5.397573

Cu_4

Cu 6.463658 7.592496 6.348217

Cu 7.808087 6.673994 7.922681

Cu 5.455040 6.389678 8.119416

Cu 8.812601 7.883219 6.149215

Cu_5

Cu 7.198979 7.897425 6.947613

Cu 9.113454 7.935263 8.370246

Cu 7.312316 6.454341 8.846205

Cu 5.450883 6.429358 7.405559

Cu 8.979010 9.338256 6.484868

Cu_6

Cu 7.814696 7.191311 8.989532

Cu 7.804375 9.559688 8.581551

Cu 9.287198 8.143649 7.326582

Cu 6.382145 8.600456 10.181418

Cu 9.292869 5.852095 7.778153

Cu 9.212276 10.446359 6.936158

Cu_7

Cu	6.904564	6.834542	6.892011
Cu	8.109850	8.710585	5.933099
Cu	8.327622	8.382541	8.331236
Cu	8.151340	6.002830	8.777419
Cu	6.209927	9.095765	7.381691
Cu	6.250438	7.452527	9.153626
Cu	9.325615	6.800568	6.810121

Cu_8

Cu	8.986183	7.901047	6.833536
Cu	6.956532	6.790087	8.830604
Cu	7.514671	9.108641	8.349815
Cu	6.948049	6.631660	6.399882
Cu	7.085041	9.016752	5.964860
Cu	5.433768	8.109148	7.588584
Cu	9.184417	7.626259	9.213500
Cu	8.736497	5.661410	7.664224

Cu_9

Cu	7.340215	9.007876	9.001348
Cu	9.479141	7.992725	9.617893
Cu	9.230247	7.130970	7.364004
Cu	8.619675	9.436752	6.944281
Cu	6.904983	7.756266	6.969079
Cu	9.310790	10.358687	9.075630
Cu	7.597597	6.600091	9.093480
Cu	10.839793	8.897932	7.822035
Cu	5.405368	7.546346	8.840059

Cu_{10}

Cu	8.732306	6.835625	8.237733
Cu	7.332896	8.574455	9.458672
Cu	9.550911	9.162413	8.547917
Cu	7.615428	8.662817	6.989591
Cu	7.710197	10.678343	8.279658
Cu	9.868765	8.051232	6.444492
Cu	9.307490	7.613330	10.424932
Cu	6.342928	6.883595	8.075556
Cu	9.285066	10.425567	6.419300
Cu	7.331696	6.190474	10.200002

Cu_{11}

Cu	8.926787	10.149770	10.545526
Cu	9.550906	8.699966	8.347810
Cu	11.272082	9.898726	9.706934
Cu	10.084444	8.037766	10.753239
Cu	9.681957	11.120132	8.308724
Cu	7.515284	10.102232	8.587830
Cu	7.824519	8.011678	9.890715
Cu	11.587147	7.550567	8.951941
Cu	10.337975	12.097921	10.401473
Cu	8.018551	12.262940	9.675667
Cu	9.484194	6.352335	9.114554

Cu_{12}

Cu	8.842829	7.705579	7.142196
Cu	8.051778	8.941769	9.412142
Cu	9.537713	10.006445	7.647011
Cu	7.146899	9.523237	7.183060
Cu	10.430611	8.200459	8.947860
Cu	6.609330	7.380625	8.223801

Cu 8.628017 6.547884 9.338518

Cu 7.949347 11.267946 8.897580

Cu 9.889462 10.365122 10.090466

Cu 9.513066 8.192434 11.149105

Cu 6.793188 7.456635 5.858087

Cu 7.739802 5.544245 7.242552

Cu_{13}

Cu 9.892563 10.432230 10.415316

Cu 7.763274 9.290822 10.732983

Cu 8.795929 8.841647 8.490454

Cu 10.110738 10.820367 7.940300

Cu 7.916274 11.101089 9.058066

Cu 9.824489 8.012490 10.725910

Cu 11.211205 8.838722 8.819550

Cu 9.912790 6.695182 8.744427

Cu 9.890621 12.640721 9.463675

Cu 11.961223 11.196013 9.461414

Cu 11.968381 9.301764 11.023071

Cu 6.422503 9.080762 8.765997

Cu 7.676719 7.094514 9.705730

9.2 monometallic In

In_2

In 4.989480 6.518878 6.337119

In 8.032296 6.502897 6.684657

In_3

In 7.511602 5.959793 6.102926

In 7.231444 6.545740 8.988865

In 6.495615 8.732987 6.146729

In_4

In	5.352972	6.779522	5.372591
In	8.340879	6.917081	8.327846
In	8.327630	6.974476	5.353073
In	5.371949	6.722488	8.339921

In_5

In	7.533421	6.662819	5.273175
In	5.224335	5.288969	6.259279
In	6.107087	8.975376	6.438303
In	8.260044	5.559882	7.938550
In	7.644207	8.282045	8.859645

In_6

In	8.428304	8.717191	8.454348
In	6.162333	5.229131	7.704169
In	5.629820	7.364484	5.556087
In	9.023489	5.839461	7.575489
In	5.562878	8.128155	8.530818
In	8.484450	8.012854	5.470509

In_7

In	5.621952	6.500730	5.229103
In	5.132751	8.719536	7.734347
In	8.052620	8.265053	8.481554
In	8.545257	5.552680	5.306298
In	8.180593	5.357309	8.256928
In	7.778212	8.517462	5.616838
In	5.291367	5.689844	7.977684

In_8

In	8.626317	5.746696	7.030624
In	6.021389	6.543873	8.689192
In	9.013803	8.484396	6.327822

In	6.411548	9.286676	8.000988
In	5.825763	5.785412	5.702777
In	8.804443	6.486727	10.039043
In	6.213342	8.550667	5.011951
In	9.210299	9.242606	9.324507

In_9

In	9.490917	7.171718	9.909079
In	9.252891	10.035721	10.084566
In	8.776847	7.686893	6.868890
In	8.497164	10.628724	6.975592
In	11.714684	7.801421	7.884249
In	6.366366	9.932391	8.985980
In	11.356661	10.704850	7.645467
In	6.877472	7.923005	11.757025
In	6.550981	6.998908	8.772608

In_{10}

In	7.647586	8.231854	7.967769
In	9.929558	9.459976	9.610306
In	9.968578	9.729768	6.333670
In	8.639545	5.597300	8.675244
In	7.524181	7.662233	11.376205
In	10.873381	7.105953	7.251299
In	6.702498	10.995543	7.412628
In	6.938279	10.347057	10.372381
In	10.407435	6.859121	10.795376
In	9.398504	12.040387	8.234492

In_{11}

In	9.518426	10.324837	10.123768
In	9.680846	11.915770	7.435841
In	12.319447	9.096717	10.776819

In 10.674543 6.662583 10.112123

In 10.686481 8.587620 7.734324

In 9.302524 8.146346 12.348888

In 6.719634 9.545489 11.675447

In 7.964813 7.735612 9.170473

In 7.970413 9.544618 6.699736

In 6.670786 10.961996 9.018353

In 12.328264 11.315155 8.740971

In_{12}

In 10.356497 9.614732 9.117774

In 7.571825 8.479424 7.827100

In 9.539539 10.204348 12.176009

In 7.898454 11.877663 9.855897

In 12.664523 10.583402 10.764551

In 10.516456 12.856517 10.937304

In 9.421556 7.162820 10.569080

In 8.847193 11.141430 7.007619

In 11.351142 12.551132 8.068724

In 11.587334 8.229583 12.584348

In 7.027449 9.148776 10.709278

In 12.553708 7.486046 9.717992

In_{13}

In 9.702015 9.551982 9.525513

In 8.285606 9.632379 12.374104

In 6.790883 8.825245 8.210742

In 10.692340 6.668478 9.016870

In 9.571223 8.247823 6.718760

In 11.748637 11.036462 7.730711

In 8.619363 11.268381 7.188380

In 9.907275 12.819933 9.683905

In 7.200899 11.289495 10.121425

In 12.768139 9.311827 9.960263

In 10.976190 11.198323 11.926492

In 7.806964 7.172817 10.466138

In 10.795135 7.840953 11.941176

9.3 Bimetallic CuIn

Cu_1In_1

Cu 7.651290 7.348905 7.164705

In 5.828640 5.974576 6.062569

Cu_2In_1

Cu 6.685096 7.082710 8.923226

Cu 7.053747 6.004655 6.914204

In 8.197564 8.375229 7.177567

Cu_3In_1

Cu 7.639879 8.814580 9.317575

Cu 8.537016 8.559238 7.208114

Cu 7.879998 6.965965 5.651080

In 6.520595 6.892081 7.894382

Cu_4In_1

Cu 7.348486 6.083352 7.186931

Cu 8.535547 7.315083 8.893275

Cu 6.157151 7.320678 8.826563

Cu 9.482580 7.030738 6.732199

In 7.411314 8.779751 6.909799

Cu_5In_1

Cu 7.229953 5.880531 7.120104

Cu 8.514443 6.719747 8.946537

Cu 6.273062 7.509097 8.752553

Cu 9.200822 7.375304 6.738945

Cu 8.267811 9.010839 8.372402

In 6.799754 8.322939 6.329579

Cu_6In_1

Cu 8.739849 9.666272 9.558608

Cu 9.775466 7.666366 8.709465

Cu 6.907495 9.432294 7.888169

Cu 9.208667 9.523363 7.199123

Cu 7.970572 7.424060 7.055526

Cu 10.284433 7.600967 6.330077

In 7.260041 7.404910 9.633493

Cu_7In_1

Cu 9.355155 9.871283 8.511221

Cu 9.310617 7.519888 7.652948

Cu 8.611247 8.008307 10.011095

Cu 7.748408 9.164455 6.871526

Cu 7.022299 7.325280 8.350359

Cu 7.067056 9.658797 9.197499

Cu 5.457232 8.974927 7.561936

In 11.216344 8.162235 9.400073

Cu_8In_1

Cu 10.425940 11.767112 11.170863

Cu 10.462825 9.531306 9.607965

Cu 11.501323 11.586364 8.883804

Cu 9.083245 11.578415 9.189165

Cu 12.477145 10.559839 10.827266

Cu 8.681855 10.107161 11.148388

Cu 10.390749 13.599391 9.447351

Cu 12.437015 13.002340 10.638994

In 10.977709 8.837186 12.205559

Cu_9In_1

Cu 7.184081 7.495778 6.555281

Cu 8.509089 7.856502 8.862650

Cu 5.974418 8.029150 8.574942

Cu 9.173899 6.237570 7.181690

Cu 7.451300 9.726664 7.749169

Cu 9.313503 8.626310 6.611082

Cu 5.423252 9.163229 6.474692

Cu 7.533748 9.716001 5.391730

Cu 9.525296 5.736639 9.495863

In 6.981059 5.551236 8.838419

9.4 Bimetallic InCu

In_1Cu_2

In 8.197564 8.375229 7.177567

Cu 6.685096 7.082710 8.923226

Cu 7.053747 6.004655 6.914204

In_2Cu_1

In 6.672437 6.336367 5.007273

In 6.047325 6.384568 8.155671

Cu 7.837217 7.829144 6.860411

In_2Cu_2

In 6.203395 8.484751 7.780577

In 8.980477 8.054698 6.772045

Cu 7.203024 6.158090 7.111452

Cu 8.111798 7.301998 9.121376

In_2Cu_3

In 9.230536 8.268092 7.922586

In 6.103728 6.975080 8.318351

Cu 7.427957 7.424901 5.893044

Cu 8.317314 5.833357 7.403261

Cu 6.868387 9.280911 7.256642

In_3Cu_2

In 6.535330 6.711805 8.350669

In 8.018749 9.830102 10.288125

In 9.702989 7.918051 6.488837

Cu 9.048641 7.654013 9.085268

Cu 9.971640 9.847281 8.396459

Cu 7.673783 9.180279 7.701315

In_3Cu_1

In 8.438763 7.191957 9.046842

In 6.139115 8.816400 7.854386

In 8.844342 7.077279 6.010446

Cu 6.690469 6.188295 7.407394

In_4Cu_1

In 6.206058 5.401229 5.391613

In 6.916390 5.376496 8.401658

In 6.364531 8.353860 5.390320

In 7.077645 8.328688 8.385630

Cu 8.344887 6.761091 6.496163

In_5Cu_1

In 7.090417 6.753731 5.934583

In 9.033523 8.821525 7.072795

In 8.399522 7.883911 9.840535

In 6.248882 9.674590 6.280460

In 6.504069 5.776241 8.695250

Cu 9.038425 6.147098 7.879073

In_6Cu_1

In 8.693503 8.140447 9.537285

In 8.669438 9.544365 6.968032

In 8.546251 5.450099 8.104392

In 5.866376 6.720417 8.785918

In 5.834157 8.118958 6.217213

In 8.537626 6.895876 5.501473

Cu 6.472644 9.348705 8.553404

In_7Cu_1

In 8.582129 6.536089 6.606877

In 8.636930 9.852283 7.162237

In 8.477489 6.087296 9.514285

In 8.491298 9.384789 10.016451

In 6.152762 8.294163 6.798034

In 11.682559 9.992912 8.812598

In 6.017618 7.821856 9.649496

Cu 10.073846 7.869269 8.357991

In_8Cu_1

In 8.519061 5.929688 9.896825

In 8.801198 7.410859 6.793796

In 7.910283 9.068963 9.935282

In 8.408298 10.457850 7.057596

In 6.140663 8.716888 5.681605

In 6.102787 6.903986 8.258402

In 10.863045 9.546804 8.697187

In 11.222297 6.559900 8.720421

Cu 10.153142 7.921645 10.748580

In_9Cu_1

In 9.975029 8.902237 9.268831

In 6.822640 7.787789 7.752378

In 9.783240 7.726488 6.387178

In 7.614830 10.797335 8.673216

In 9.094826 5.648501 8.535097

In 6.863311 8.751091 10.845781

In 8.951028 6.664914 11.363998

In 10.154225 10.829216 6.760290

In 7.509020 9.825197 5.663932

Cu 6.786511 6.206577 9.851397

References:

(1) van Albada, G. A.; Mutikainen, I.; Turpeinen, U.; Reedijk, J. Crystal Structure, Magnetism and Spectroscopy of a Dinuclear Cu (II) Compound with a Chiral Ligand; Cu2 (SL)(μ-Cl)(Cl) 2 (CH3OH)(HSL= S-1, 2-Bis (Benzimidazol-2-Yl)-1-Hydroxyethane). *Polyhedron* **2006**, *25* (1), 81–86.

(2) Zumdick, M. F.; Landrum, G. A.; Dronskowski, R.; Hoffmann, R.-D.; Pöttgen, R. Structure, Chemical Bonding, and Properties of ZrIn2, IrIn2, and Ti3Rh2In3. *Journal of Solid State Chemistry* **2000**, *150* (1), 19–30.

(3) Ahmed, A. A. Structural and Electronic Properties of the Adsorption of Nitric Oxide Molecule on Copper Clusters CuN (N= 1–7): A DFT Study. *Chemical Physics Letters* **2020**, *753*, 137543.

(4) Hakkar, F.; Zouchoune, B. Predicted Structures and Electronic Properties of Gallium-Indium Clusters GamInn–m (n = 4, 6, 8 and m < n): A Density Functional Study. *J Struct Chem* **2018**, *59* (5), 997–1009. https://doi.org/10.1134/S0022476618050013.
